# Causal Effects of Gut Microbiome on Systemic Lupus Erythematosus: A Two-Sample Mendelian Randomization Study

**DOI:** 10.3389/fimmu.2021.667097

**Published:** 2021-09-07

**Authors:** Kun Xiang, Peng Wang, Zhiwei Xu, Yu-Qian Hu, Yi-Sheng He, Yue Chen, Ya-Ting Feng, Kang-Jia Yin, Ji-Xiang Huang, Jie Wang, Zheng-Dong Wu, Xiao-Ke Yang, De-Guang Wang, Dong-Qing Ye, Hai-Feng Pan

**Affiliations:** ^1^Department of Epidemiology and Biostatistics, School of Public Health, Anhui Medical University, Hefei, China; ^2^Inflammation and Immune Mediated Diseases Laboratory of Anhui Province, Hefei, China; ^3^Center for Genetic Epidemiology and Genomics, School of Public Health, Soochow University Medical College, Suzhou, China; ^4^School of Public Health, Faculty of Medicine, University of Queensland, Brisbane, QLD, Australia; ^5^Department of Rheumatology and Immunology, The First Affiliated Hospital of Anhui Medical University, Hefei, China; ^6^Department of Nephrology, Second Affiliated Hospital of Anhui Medical University, Hefei, China

**Keywords:** autoimmune disease, Mendelian randomization, gut microbiome, systemic lupus erythematosus, causality

## Abstract

The observational association between gut microbiome and systemic lupus erythematosus (SLE) has been well documented. However, whether the association is causal remains unclear. The present study used publicly available genome-wide association study (GWAS) summary data to perform two-sample Mendelian randomization (MR), aiming to examine the causal links between gut microbiome and SLE. Two sets of MR analyses were conducted. A group of single nucleotide polymorphisms (SNPs) that less than the genome-wide statistical significance threshold (5 × 10^-8^) served as instrumental variables. To obtain a comprehensive conclusion, the other group where SNPs were smaller than the locus-wide significance level (1 × 10^-5^) were selected as instrumental variables. Based on the locus-wide significance level, the results indicated that there were causal effects of gut microbiome components on SLE risk. The inverse variance weighted (IVW) method suggested that *Bacilli* and *Lactobacillales* were positively correlated with the risk of SLE and *Bacillales*, *Coprobacter* and *Lachnospira* were negatively correlated with SLE risk. The results of weighted median method supported that *Bacilli*, *Lactobacillales*, and *Eggerthella* were risk factors for SLE and *Bacillales* and *Coprobacter* served as protective factors for SLE. The estimates of MR Egger suggested that genetically predicted *Ruminiclostridium6* was negatively associated with SLE. Based on the genome-wide statistical significance threshold, the results showed that *Actinobacteria* might reduce the SLE risk. However, Mendelian randomization pleiotropy residual sum and outlier (MR-PRESSO) detected significant horizontal pleiotropy between the instrumental variables of *Ruminiclostridium6* and outcome. This study support that there are beneficial or detrimental causal effects of gut microbiome components on SLE risk.

## 1 Introduction

Systemic lupus erythematosus (SLE) is an autoimmune connective tissue disease involving multiple organs, and it presents with a range of clinical symptoms, including skin rash, pericarditis, nephritis, and neurological and hematological involvement. Loss of tolerance to autoantigens is one of the hallmarks of SLE. Genetic, hormonal, and environmental factors interact in susceptible individuals, resulting in autoantibodies deposition and abnormal production of proinflammatory cytokines (1). In addition, ultraviolet light and infections induce DNA damage and apoptosis which increase exposure to autoantigens are potential triggers for SLE as well (2). The current treatment strategy is mainly the use of non-selective immunosuppressive agents. Long-term use of immunosuppressants weakens the immunity and results in severe infections (3). Therefore, it is imperative to explore the etiology of SLE to facilitate the development of treatment strategies with low damage or even no side effects.

Recently, the causal link between the gut microbiome composition and SLE risk has attracted widespread attention. The intestinal microbiota plays a critical role in the maturation of the host immune response and provide protection against pathogen overgrowth (4). A study demonstrated that the gut microbiome was related to the dynamics of human immune cells, suggesting that the gut microbiome drove the modulation of the immune system (5). The dysbiosis of gut microbiome affected immune responses, which contributed to the occurrence of autoimmune diseases (6). One possible explanation was that the presence of commensal gut microbiome influenced the autoimmune responses to nuclear antigens (7). Several studies indicated that SLE patients had dysbiosis of gut microbiome and decreased species richness (8, 9). Furthermore, the decrease in species diversity was particularly significant in patients with high SLE activity index (10), suggesting that intestinal flora might be involved in the immune pathogenesis of autoimmune diseases. Nevertheless, it remains unclear as to whether there is a causal relationship between gut microbiome and SLE.

Mendelian randomization (MR) is an approach integrating summary data of genome-wide association study (GWAS), and hence, the impact of confounding factors (e.g., environment) is minimized. MR is a common method to infer whether there are causal relationships between exposure and complex outcomes. Genetic variants that are significantly related to exposure are selected as instrumental variables to infer the causality (11). The instrumental variables that affect the exposure will affect the results proportionally if the exposure is causal. In the current study, the two-sample MR was conducted to examine if there is a causal relationship between gut microbiome composition and SLE risk.

## 2 Materials and Methods

### 2.1 Data Sources and SNP Selection

Single-nucleotide polymorphisms (SNPs) related to human gut microbiome composition were selected as instrumental variables from a GWAS with 18,473 individuals, including 122,110 variant sites (12). It was a multi-ethnic large-scale GWAS that recruited 25 population-based cohorts from the United States, Canada, Israel, the Netherlands, Belgium, Sweden, South Korea, Germany, Denmark, Finland, and the UK to explore the association between autosomal human genetic variants and the gut microbiome. Effect estimates of the SNPs related to SLE risk were extracted from a large SLE GWAS, which involved 7,219 cases and 15,991 controls of European ancestry (13).

To ensure the authenticity and accuracy of the conclusions on the causal link between gut microbiome and SLE risk, the following quality control steps were used to select optimal instrument variables. First, SNPs significantly related to gut microbiome were selected as instrumental variables. Two thresholds were used to select the instrumental variable. A set of SNPs less than the genome-wide statistical significance threshold (5 × 10^-8^) served as instrumental variables. In order to obtain more comprehensive results, the other group where SNPs are smaller than the locus-wide significance level (1 × 10^-5^) was selected as instrumental variables. Second, the minor allele frequency (MAF) threshold of variants of interest was 0.01. Third, one of the principles of the MR approach is that there is no linkage disequilibrium (LD) among the included instrumental variables, since the presence of strong LD might result in biased results. In the current study, the clumping process (*R* ^2^ < 0.001 and clumping distance = 10,000kb) were conducted to assess the LD between the included SNPs. Fourth, an important step of MR is to ensure that the effects of the SNPs on the exposure correspond to the same allele as the effects on the outcome. In accordance with the principle, palindromic SNPs would not be included in the instrumental variables. Fifth, when SNPs related to exposure were absent in the outcome GWAS, the proxy SNPs significantly associated with the variants of interest were selected (*r* ^2^ > 0.8).

### 2.2 The Assumptions of MR

To minimize the impact of bias on the results, the MR method must conform to three important assumptions. First, instrumental variables are independent of confounders that influence exposure and outcome. Second, the variants of interest used in the analysis should be significantly associated with exposure. *F* statistic is generally performed to assess the strength of the relevance between instrumental variables and exposure. The formula of *F* statistic is *F* = *R* ^2^(*n*-*k*-1)/*k*(1-*R* ^2^). *R* ^2^ represents the exposure variance explained by the selected SNPs, *n* is the sample size, and *k* represent the number of included instrumental variables. If *F* is less than 10, there is a weak association between instrumental variables and exposure. Third, instrumental variables affect outcomes only through exposure, which means that there is no horizontal pleiotropy effect between instrumental variables and outcome.

### 2.3 MR Estimates

In the current study, high-efficiency methods including inverse variance weighted (IVW), MR-Egger, weighted median, and weighted mode were used to infer whether there was causal effect of human gut microbiome composition on SLE risk. IVW is essentially a meta-analysis method, which converts to a weighted regression of the outcome effects of instrumental variables on the exposure effects to obtain an overall estimate of the impact of gut microbiome on the risk of SLE, where the intercept is limited to zero (14). When there is no horizontal pleiotropy, IVW can avoid the impact of confounding factors to obtain unbiased estimates. MR-Egger may be strongly influenced by outlying genetic variables, leading to inaccurate estimates. However, even if all selected instrumental variables are invalid, the MR-Egger method can still provide unbiased estimates. The weighted median can provide consistent estimates of the causal effects, even if as many as 50% of the information in the analysis comes from variations of interest are invalid instrumental variables. The weighted median method has some important advantages over the MR-Egger since it improves the accuracy of the results. When most instrumental variables with similar causal estimates are valid, the weighted mode approach is still valid even if the other instrumental variables do not meet the requirements of MR method for causal inference (15).

The MR-Egger regression was conducted to assess whether the included SNPs had potential horizontal pleiotropic effects. MR-Egger regression is a method, which has the property that both detect and adjust for pleiotropy in the MR analysis, and get a causal effect estimate (16) and examine whether the results are driven by the directional horizontal pleiotropy (17). Given the lower accuracy and statistical power of MR-Egger regression, Mendelian randomization pleiotropy residual sum and outlier (MR-PRESSO) was performed to detect any outliers reflecting likely pleiotropic biases and correct horizontal pleiotropy. Furthermore, Cochran’s *Q* statistic was used to quantify the heterogeneity among the selected SNPs. To determine whether there were potential strong influence SNPs, the leave-one-out sensitivity analysis was performed to verify the reliability and stability of the causal effect estimates. Statistical analyses were performed using R software (version 4.0.2, TwoSampleMR package).

## 3 Results

### 3.1 Instrumental Variables Selection

Initially, 14,587 (locus-wide significance level, *P* < 1 × 10^-5^) and 456 (genome-wide statistical significance threshold, *P* < 5 × 10^-8^) SNPs were identified as instrumental variables from a large-scale GWAS. It contained 211 bacterial traits, including five biological classifications: phylum, class, order, family, and genus. After removing SNPs that had LD effects and independence from SLE, 2,105 (*P* < 1 × 10^-5^) and 13 (*P* < 5 × 10^-8^) SNPs were selected as instrumental variables. The main information of SNPs including effect allele, other allele, beta, SE, and *P* value were collected systematically for further analysis.

### 3.2 Two-Sample MR Analysis

#### 3.2.1 Locus-Wide Significance Level

The results of IVW analyses demonstrated that *Bacilli* (odds ratio (OR) = 1.40, 95% confidence interval (CI), 1.02–1.93, *P* = 0.037) and *Lactobacillales* (OR = 1.40, 95% CI, 1.01–1.95, *P* = 0.045) were positively correlated with the risk of SLE and *Bacillales* (OR = 0.85, 95% CI, 0.74–0.98, *P* = 0.022), *Coprobacter* (OR = 0.78, 95% CI, 0.64–0.95, *P* = 0.014), and *Lachnospira* (OR = 0.60, 95% CI, 0.38–0.94, *P* = 0.027) were negatively correlated with SLE risk (**Table 1**). The MR estimates of weighted median indicated that *Bacilli* (OR = 1.59, 95% CI, 1.06–2.39, *P* = 0.027), *Lactobacillales* (OR = 1.73, 95% CI, 1.13–2.64, *P* = 0.011), and *Eggerthella* (OR = 1.41, 95% CI, 1.05–1.90, *P* = 0.022) were risk factors for SLE, and *Bacillales* (OR = 0.81, 95% CI, 0.67–0.96, *P* = 0.018) and *Coprobacter* (OR = 0.77, 95% CI, 0.59–0.99, *P* = 0.043) served as protective factors for SLE (**Table 1**). The estimates of MR Egger suggested that genetically predicted *Ruminiclostridium6* were negatively associated with SLE (OR = 0.35, 95% CI, 0.15–0.83, *P* = 0.040). The detailed statistical results of the 211 intestinal floras were shown in **Supplementary Table S1**.

**Table 1:** MR results of causal links between gut microbiome and SLE risk (*P* < 1 × 10^-5^).

Classification		Nsnp	Methods	Beta	SE	OR (95% CI)	*P* value	Horizontal pleiotropy			Heterogeneity		*F* statistic
								Egger intercept	SE	*P* value	Cochran’s *Q*	*P* value	
Class	*Bacilli*	16	MR Egger	0.61	0.44	1.84 (0.77–4.38)	0.189	-0.02	0.03	0.519	19.63	0.142	24.73
			Weighted median	0.46	0.21	1.59 (1.06–2.39)	0.027						
			Inverse variance weighted	0.34	0.16	1.40 (1.02–1.93)	0.037						
			Weighted mode	0.65	0.34	1.91 (0.98–3.75)	0.078						
Order	*Lactobacillales*	14	MR Egger	0.22	0.44	1.24 (0.52–2.94)	0.634	0.01	0.03	0.769	16.71	0.161	25.54
			Weighted median	0.55	0.22	1.73 (1.13–2.64)	0.011						
			Inverse variance weighted	0.34	0.17	1.40 (1.01–1.95)	0.045						
			Weighted mode	0.57	0.33	1.78 (0.93–3.34)	0.107						
	*Bacillales*	11	MR Egger	0.07	0.30	1.08 (0.60–1.95)	0.813	-0.03	0.04	0.448	7.62	0.666	24.69
			Weighted median	-0.22	0.09	0.81 (0.67–0.96)	0.018						
			Inverse variance weighted	-0.16	0.07	0.85 (0.74–0.98)	0.022						
			Weighted mode	-0.28	0.14	0.76 (0.57–1.01)	0.080						
Genus	*Coprobacter*	12	MR Egger	-0.01	0.38	0.99 (0.47–2.08)	0.975	-0.03	0.04	0.533	12.35	0.262	26.60
			Weighted median	-0.26	0.13	0.77 (0.59–0.99)	0.043						
			Inverse variance weighted	-0.25	0.10	0.78 (0.64–0.95)	0.014						
			Weighted mode	-0.40	0.24	0.67 (0.42–1.07)	0.121						
	*Eggerthella*	10	MR Egger	0.55	0.70	1.73 (0.44–6.79)	0.453	-0.03	0.08	0.675	19.35	0.022	22.42
			Weighted median	0.35	0.15	1.41 (1.05–1.90)	0.022						
			Inverse variance weighted	0.25	0.15	1.29 (0.95–1.74)	0.097						
			Weighted mode	0.36	0.18	1.43 (0.99–2.06)	0.085						
	*Lachnospira*	7	MR Egger	-0.31	1.11	0.73 (0.08–6.48)	0.790	-0.01	0.07	0.861	8.21	0.145	10.83
			Weighted median	-0.49	0.26	0.61 (0.37–1.02)	0.059						
			Inverse variance weighted	-0.51	0.23	0.60 (0.38–0.94)	0.027						
			Weighted mode	-0.49	0.37	0.61 (0.30–1.26)	0.231						
	*Ruminiclostridium6*	11	MR Egger	-1.04	0.43	0.35 (0.15–0.83)	0.040	0.10	0.05	0.065	13.11	0.158	23.83
			Weighted median	-0.37	0.22	0.69 (0.45–1.08)	0.102						
			Inverse variance weighted	-0.20	0.19	0.82 (0.56–1.19)	0.293						
			Weighted mode	-0.47	0.33	0.63 (0.33–1.21)	0.193						

SLE, systemic lupus erythematosus; SNP, single nucleotide polymorphism; OR, odds ratio.

**Figure 1:**
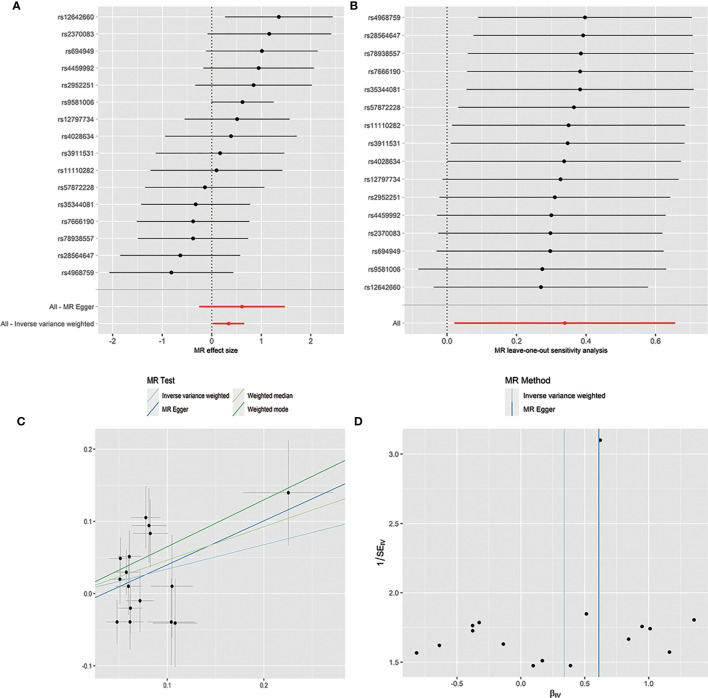
Forest plot **(A)**, sensitivity analysis **(B)**, scatter plot **(C)**, and funnel plot **(D)** of the causal effect of *Bacilli* on SLE risk.

The horizontal pleiotropy between instrumental variables and outcome was assessed by MR-Egger regression, and the results showed that there was no evidence of horizontal pleiotropy (**Table 1**). No outliers were found in the analysis of *Bacilli* (*P* = 0.191), *Lactobacillales* (*P* = 0.213), *Bacillales* (*P* = 0.403), *Coprobacter* (*P* = 0.365), and *Lachnospira* (*P* = 0.301) by MR-PRESSO. MR-PRESSO suggested that there was significant horizontal pleiotropy between the instrumental variables of *Eggerthella* and outcome (*P* = 0.041), and rs1784446 was identified as outlier. However, the results did not change significantly after removing the SNP (OR = 1.42, 95% CI, 1.06–1.90, *P* = 0.020). In the analysis of *Ruminiclostridium6*, MR-PRESSO found there was significant horizontal pleiotropy (*P* = 0.045) and rs61060922 was identified as a pleiotropic SNP. After removing the outlier, the results changed substantially (OR = 0.48, 95% CI, 0.22–1.04, *P* = 0.101). The detailed information of the instrumental variables was shown in **Table 2**. The *F* statistics of the SNPs were all greater than 10, indicating that there was no weak instrumental variables bias (**Table 1**). Thus, the two-sample MR estimates found that *Bacilli* (**Figure 1**), *Eggerthella* (**Supplementary Figure S1**), and *Lactobacillales* (**Supplementary Figure S2**) were positively related to SLE risk, and *Coprobacter* (**Supplementary Figure S3**), *Bacillales* (**Supplementary Figure S4**), and *Lachnospira* (**Supplementary Figure S5**) played protective roles in the pathogenesis of SLE.

**Table 2:** SNPs used as instrumental variables from gut microbiome and SLE GWASs (*P* < 1 × 10^-5^).

Bacterial traits	SNP	Effect allele	Other allele	Gut microbiome			SLE			Proxy SNP	Target effect allele	Target other allele
				Beta	SE	*P* value	Beta	SE	*P* value			
*Bacilli*	rs11110282	A	G	-0.10	0.02	2.08E-06	-0.01	0.07	0.888	–	–	–
	rs12642660	A	G	-0.08	0.02	8.32E-07	-0.11	0.04	0.014	–	–	–
	rs12797734	C	T	0.06	0.01	5.58E-06	0.03	0.03	0.344	–	–	–
	rs2370083	G	T	-0.08	0.02	5.98E-06	-0.09	0.05	0.068	–	–	–
	rs28564647	G	T	-0.06	0.02	5.98E-06	0.04	0.04	0.301	–	–	–
	rs2952251	G	A	-0.06	0.01	6.59E-07	-0.05	0.04	0.160	–	–	–
	rs35344081	A	G	0.06	0.01	9.35E-07	-0.02	0.03	0.560	–	–	–
	rs3911531	C	T	-0.06	0.01	8.53E-06	-0.01	0.04	0.800	–	–	–
	rs4028634	C	T	0.05	0.01	3.08E-06	0.02	0.03	0.568	–	–	–
	rs4459992	C	T	0.05	0.01	9.31E-06	0.05	0.03	0.096	–	–	–
	rs4968759	A	G	-0.05	0.01	9.14E-06	0.04	0.03	0.201	–	–	–
	rs57872228	C	T	-0.07	0.01	7.28E-07	0.01	0.04	0.821	–	–	–
	rs694949	G	A	-0.08	0.02	5.00E-06	-0.08	0.05	0.078	–	–	–
	rs7666190	A	C	-0.10	0.02	7.60E-06	0.04	0.06	0.515	–	–	–
	rs78938557	C	T	0.11	0.02	9.54E-07	-0.04	0.06	0.505	–	–	–
	rs9581006	C	T	0.23	0.05	1.80E-06	0.14	0.07	0.054	–	–	–
*Lactobacillales*	rs11110282	A	G	-0.11	0.02	1.64E-06	-0.01	0.07	0.888	–	–	–
	rs11627423	A	C	0.05	0.01	8.94E-06	0.03	0.03	0.374	–	–	–
	rs11639594	A	C	0.05	0.01	9.53E-06	0.03	0.03	0.294	–	–	–
	rs12642660	A	G	-0.07	0.02	2.42E-06	-0.11	0.04	0.014	–	–	–
	rs12797734	C	T	0.06	0.01	6.07E-06	0.03	0.03	0.344	–	–	–
	rs2370083	G	T	-0.08	0.02	3.84E-06	-0.09	0.05	0.068	–	–	–
	rs2952251	G	A	-0.06	0.01	2.00E-07	-0.05	0.04	0.160	–	–	–
	rs34989881	A	G	0.11	0.02	7.26E-06	-0.11	0.06	0.081	–	–	–
	rs35344081	A	G	0.06	0.01	3.70E-07	-0.02	0.03	0.560	–	–	–
	rs4028634	C	T	0.05	0.01	1.89E-06	0.02	0.03	0.568	–	–	–
	rs57872228	C	T	-0.07	0.01	2.08E-06	0.01	0.04	0.821	–	–	–
	rs78938557	C	T	0.11	0.02	2.01E-06	-0.04	0.06	0.505	–	–	–
	rs7919839	C	T	0.08	0.02	8.97E-06	-0.01	0.05	0.846	–	–	–
	rs9581006	C	T	0.23	0.05	1.77E-06	0.14	0.07	0.054	–	–	–
*Bacillales*	rs10410917	C	T	0.11	0.02	5.50E-06	-0.03	0.03	0.259	–	–	–
	rs821056	A	G	0.21	0.04	7.43E-06	-0.01	0.04	0.789	–	–	–
	rs11608727	G	T	0.13	0.03	6.17E-06	0.05	0.03	0.127	–	–	–
	rs12498725	A	G	-0.18	0.04	1.78E-06	0.01	0.04	0.782	–	–	–
	rs182923	T	C	0.14	0.03	4.61E-06	-0.05	0.04	0.214	–	–	–
	rs4617108	A	G	0.25	0.05	1.04E-06	-0.05	0.06	0.377	–	–	–
	rs55793055	C	T	-0.11	0.03	7.79E-06	0.04	0.03	0.126	–	–	–
	rs62141894	A	G	0.14	0.03	6.90E-06	-0.02	0.03	0.556	–	–	–
	rs62640857	A	G	0.15	0.03	3.58E-06	-0.04	0.04	0.346	–	–	–
	rs7611581	G	T	-0.11	0.02	3.00E-06	0.03	0.02	0.231	–	–	–
	rs875142	A	G	0.13	0.03	5.13E-06	-0.04	0.03	0.181	–	–	–
*Coprobacter*	rs11532348	C	T	-0.10	0.02	4.34E-06	-0.01	0.03	0.753	–	–	–
	rs12684609	C	T	0.10	0.02	5.70E-06	-0.02	0.03	0.487	–	–	–
	rs12996055	A	C	0.09	0.02	5.03E-06	-0.04	0.03	0.229	–	–	–
	rs143180826	C	T	0.17	0.04	6.60E-06	0.05	0.06	0.431	–	–	–
	rs143662916	C	T	0.25	0.05	3.07E-06	-0.11	0.07	0.143	–	–	–
	rs189356	A	G	0.08	0.02	7.59E-06	-0.05	0.03	0.071	–	–	–
	rs213863	C	T	-0.09	0.02	3.78E-06	0.04	0.03	0.217	–	–	–
	rs28402691	C	T	0.11	0.02	7.14E-06	0.02	0.03	0.493	–	–	–
	rs305411	A	G	0.13	0.03	8.82E-07	-0.08	0.05	0.082	–	–	–
	rs3828477	G	T	-0.09	0.02	2.41E-06	0.04	0.03	0.220	–	–	–
	rs72821405	C	T	-0.15	0.03	4.27E-06	0.01	0.04	0.816	–	–	–
	rs74919520	A	G	0.12	0.03	6.39E-06	-0.11	0.05	0.021	–	–	–
*Eggerthella*	rs112205261	C	T	-0.19	0.04	3.48E-06	0.13	0.06	0.032	–	–	–
	rs116603267	A	G	0.16	0.04	8.39E-06	-0.07	0.06	0.221	–	–	–
	rs1784446	G	A	0.09	0.02	5.05E-06	-0.07	0.03	0.019	–	–	–
	rs2223081	G	A	0.10	0.02	3.04E-06	0.02	0.03	0.563	–	–	–
	rs2240838	A	G	0.09	0.02	1.46E-06	0.05	0.03	0.085	–	–	–
	rs3851328	G	T	-0.11	0.02	1.38E-06	0.04	0.03	0.215	–	–	–
	rs4985746	A	G	0.11	0.02	4.63E-06	-0.08	0.04	0.020	–	–	–
	rs6430926	C	T	0.09	0.02	6.10E-06	0.03	0.03	0.247	–	–	–
	rs6678488	A	C	0.09	0.02	6.20E-06	-0.02	0.04	0.587	–	–	–
	rs76663501	C	T	0.17	0.04	5.97E-06	-0.08	0.06	0.179	–	–	–
*Lachnospira*	rs13157098	A	G	-0.08	0.02	8.76E-07	-0.06	0.05	0.196	–	–	–
	rs159484	A	G	0.08	0.02	7.37E-06	-0.03	0.04	0.495	–	–	–
	rs2520509	G	A	0.05	0.01	8.52E-06	-0.06	0.03	0.032	–	–	–
	rs4686798	C	T	0.05	0.01	4.51E-06	-0.03	0.03	0.248	–	–	–
	rs4923324	A	G	-0.06	0.01	1.63E-06	0.07	0.03	0.031	–	–	–
	rs56791201	C	T	0.05	0.01	2.77E-06	-0.01	0.02	0.626	–	–	–
	rs75566846	A	C	0.12	0.03	5.88E-06	-0.11	0.07	0.152	–	–	–
*Ruminiclostridium6*	rs10829821	C	T	-0.10	0.02	4.89E-06	-0.02	0.06	0.728	rs11017525	A	G
	rs116969552	A	G	-0.17	0.04	8.01E-06	0.05	0.08	0.533	–	–	–
	rs11992182	A	C	0.06	0.01	2.69E-06	0.05	0.03	0.095	–	–	–
	rs1377110	G	A	0.06	0.01	2.20E-06	0.03	0.04	0.416	–	–	–
	rs61060922	G	T	0.16	0.03	8.98E-07	-0.19	0.06	0.002	–	–	–
	rs663262	C	T	-0.13	0.03	3.63E-06	-0.06	0.06	0.312	–	–	–
	rs67479537	C	T	0.12	0.03	5.85E-06	-0.07	0.06	0.236	–	–	–
	rs71414120	G	T	0.20	0.04	8.77E-07	-0.08	0.06	0.179	–	–	–
	rs72991535	G	T	0.14	0.03	3.42E-06	-0.07	0.07	0.306	–	–	–
	rs79968172	A	G	0.11	0.02	2.23E-06	0.07	0.06	0.244	–	–	–
	rs9555756	A	C	-0.08	0.02	3.73E-06	0.05	0.04	0.194	–	–	–

SLE, systemic lupus erythematosus; SNP, single nucleotide polymorphism.

#### 3.2.2 Genome-Wide Statistical Significance Threshold

When MR analysis was performed with gut microbiome as a whole, the results of IVW (OR = 1.20, 95% CI, 0.96–1.52, *P* = 0.114), MR Egger (OR = 0.74, 95% CI, 0.34–1.58, *P* = 0.448), weighted median (OR = 1.15, 95% CI, 0.92–1.43, *P* = 0.221), and weighted mode (OR = 1.26, 95% CI, 0.98–1.63, *P* = 0.099) showed that gut microbiome was not associated with SLE risk (**Table 3** and **Supplementary Figure S6**). The detailed information of the instrumental variables was shown in **Supplementary Table S2**. MR-Egger regression showed that there was no horizontal pleiotropy between instrumental variables and outcome (*P* = 0.213). In addition, the results of Cochrane *Q* statistics showed no significant heterogeneity (*P* = 0.052) and the *F* statistics was greater than 10. The results of gut microbiome classification indicated that *Actinobacteria* might reduce the risk of SLE (OR = 0.52, 95% CI, 0.29–0.95, *P* = 0.033) (**Table 3**). Heterogeneity and horizontal pleiotropy could not be examined due to the limited number of included SNPs.

**Table 3:** MR results of causal links between gut microbiome and SLE risk (*P* < 5 × 10^-8^).

Classification		Nsnp	Methods	Beta	SE	OR (95% CI)	*P* value	Horizontal pleiotropy			Heterogeneity		*F* statistic
								Egger intercept	SE	*P* value	Cochran’s *Q*	*P* value	
Total		13	MR Egger	-0.31	0.39	0.74 (0.34–1.58)	0.448	0.06	0.05	0.213	19.51	0.052	53.03
			Weighted median	0.14	0.11	1.15 (0.92–1.43)	0.221						
			Inverse variance weighted	0.19	0.12	1.20 (0.96–1.52)	0.114						
			Weighted mode	0.23	0.13	1.26 (0.98–1.63)	0.099						
Class	*Actinobacteria*	1	Wald ratio	-0.65	0.30	0.52 (0.29–0.95)	0.033	–	–	–	–	–	–
	*Melainabacteria*	1	Wald ratio	-0.06	0.21	0.94 (0.62–1.41)	0.766	–	–	–	–	–	–
Family	*Bifidobacteriaceae*	2	Inverse variance weighted	-0.31	0.42	0.73 (0.32–1.68)	0.459	–	–	–	–	–	–
	*Streptococcaceae*	1	Wald ratio	0.08	0.63	1.08 (0.32–3.68)	0.903	–	–	–	–	–	–
Genus	*Allisonella*	1	Wald ratio	0.34	0.18	1.40 (0.99–2.00)	0.059	–	–	–	–	–	–
	*Bifidobacterium*	2	Inverse variance weighted	-0.31	0.41	0.73 (0.33–1.65)	0.456	–	–	–	–	–	–
Order	*Bifidobacteriales*	2	Inverse variance weighted	-0.31	0.42	0.73 (0.32–1.68)	0.459	–	–	–	–	–	–

SLE, systemic lupus erythematosus; SNP, single nucleotide polymorphism; OR, odds ratio.

## 4 Discussion

This two-sample MR study suggested that the levels of *Bacillales*, *Coprobacter*, *Lachnospira*, and *Actinobacteria* were negatively related to the risk of SLE, and *Bacilli*, *Lactobacillales*, and *Eggerthella* might be the risk factors for SLE onset. However, since there were fewer instrumental variables reaching genome-wide statistical significance threshold, the results and the precision of *Actinobacteria* might have been compromised.

The gastrointestinal mucosal surface of the body is abundantly colonized by trillions of symbiotic gut microbiome which participate in the modulation and maintenance of the host immune system. Therefore, the dysbiosis of gut microbiome interacts with the intestinal mucosal immune system closely (6). Several studies found that autoimmune diseases were often accompanied by gut microbiome dysbiosis or altered microbiome. The distribution of microbes from phylum to genus levels of different taxa was different between healthy subjects and early rheumatoid arthritis (RA) patients, and the difference in microbial diversity and classification indicated that gut microbes might be involved in the pathogenesis of early RA (18). Compared with healthy controls, RA patients had varying degrees of alterations in gut microbiome composition, including *Bacteroides* (19, 20), *Prevotella* (21), *Verrucomicrobiae* (22), and *Salivarius* (23). Jangi et al. (23) found an increase in *Methanobrevibacter* and *Akkermansia* in multiple sclerosis (MS) patients, and *Methanobrevibacter* was involved in the immunomodulatory process due to its ability to recruit inflammatory cells (24). As an autoimmune disease closely related to intestinal microbes, the occurrence of inflammatory bowel disease (IBD) was often accompanied by gut microbiome dysbiosis. A study suggested that the human intestinal microbiome had an important influence on the drug metabolism and efficacy of IBD (25). Currently, there are limited studies on the association between the candidate intestinal bacteria found in this study and complex traits. Some studies indicated that compared with healthy controls, the abundance of *Bacilli* was increased in encephalitis (26) and Graves’ disease patients (27). Increased *Lactobacillales* abundance was observed in autoimmune liver disease (28), atopic dermatitis (29), type 1 diabetes (30), and Graves’ disease (27) patients. These studies indicated that the *Bacilli* and *Lactobacillales* might have the effects of promoting inflammation. The abundance of *Lachnospira* was decreased in ankylosing spondylitis (31), type 1 diabetes (32), and IgE-associated allergic disease (33) patients, and the *Lachnospira* contributed to the alleviation of inflammation in HIV-infected patients (34), suggesting that *Lachnospira* might have a protective role in inflammatory conditions. These results were consistent with the present study. However, the mechanisms by which these intestinal floras exert beneficial or detrimental effects on the immune-mediated inflammatory disease remain to be further studied.

Recently, numerous human and rodent model studies have been conducted to infer the association between SLE and gut microbiome. A study found that SLE patients, especially those in the active phase, had dysbiosis in the intestinal flora (35). Luo et al. (36) found that the microbiome of active SLE patients changed compared with the non-SLE controls and the use of non-selective immunosuppressive therapies, such as dexamethasone and azathioprine, might have a broad impact on the diversity and abundance of gut microbiome. A study indicated that primary Sjögren’s syndrome (pSS) and SLE patients shared similar alterations in the composition of gut microbiome, both showing a lower bacterial abundance and *Firmicutes*/*Bacteroidetes* ratio and a higher *Bacteroides* species richness, which could distinguish patients from individuals in the general population (37). A rodent model indicated that there were significant differences in the composition of gut microbiome between pre-disease and diseased NZB/W F1 mice, as well as between untreated group and immunosuppressive drug treatment group (36). With the progression of diseases and drug treatment, the microbiome tended to become more diverse. The fecal microbiome of SLE mice induced the production of anti-dsDNA antibodies and stimulated inflammation, and changed the expression of SLE susceptibility genes in germfree mice (38). Consistently, Choi et al. (39) demonstrated that when transferred to sterile syngeneic C57BL/6 mice, the intestinal microbes of triple congenic lupus-prone mice stimulated autoantibodies production and modulated immune cells. Intriguingly, the horizontal transfer of intestinal flora between co-bred triple congenic lupus-prone mice and syngeneic mice could mitigate the autoimmune pathogenesis.

However, even though most studies showed that SLE patients were usually accompanied by gut microbiome dysbiosis, it might only be a clinical sign of SLE and there was no causal effect on SLE risk and gut microbiome dysbiosis. First, the use of non-selective immunosuppressive agents in SLE patients could lead to alterations in gut microbiome. Second, the intestinal flora of patients with active and inactive SLE might be different, and many studies did not take into account grouping of patients. Third, the composition of gut microbiome might be different due to the inconsistency of gender ratio and ethnicities in different studies. Fourth, although studies found that SLE patients had the phenotype of gut microbiome dysbiosis, the results of changes in specific strains were not consistent. The existence of these uncertain factors obstructed the inference of the causal link between gut microbiome and SLE risk.

The main advantage of this study was that the implementation of MR approach diminished the interference of confounding factors and reverse causality on the results, which might be more convincing than observational studies. To the best of our knowledge, the study is the first MR analysis on this topic. However, some limitations should be mentioned. First, our study was unable to determine whether overlapping participants were involved in the exposure and outcome GWAS used in the two sample MR analyses. Nevertheless, the deviation from participants overlap could be minimized by the *F* statistic (40). Second, since the original research lacked demographic data (e.g., gender and race), further subgroup analysis was impossible. Third, in view of the biological plausibility and the multi-stage statistical process, applying a rigorous multiple testing correction would likely have been overly conservative, which may neglect potential strains that are causally related to SLE. Therefore, we did not account for multiple testing. Fourth, since the majority of participants in the GWAS were of European ancestry, extrapolation of the results of the study to other ethnic groups might be limited.

In summary, this MR study suggests causal effects of gut microbiome on SLE. Several types of intestinal bacteria identified in this study that potentially reduced the occurrence of SLE may have the prospects for the prevention and treatment of SLE.

## Data Availability Statement

The raw data supporting the conclusions of this article will be made available by the authors, without undue reservation.

## Author Contributions

H-FP and D-QY conceived the presented idea. KX, PW, and ZX performed the computations and manuscript writing. Y-QH, Y-SH, YC, Y-TF, K-JY, and J-XH were involved in acquisition of data. JW, Z-DW, X-KY, and D-GW were involved in interpretation of data. All authors contributed to the article and approved the submitted version.

## Conflict of Interest

The authors declare that the research was conducted in the absence of any commercial or financial relationships that could be construed as a potential conflict of interest.

## Publisher’s Note

All claims expressed in this article are solely those of the authors and do not necessarily represent those of their affiliated organizations, or those of the publisher, the editors and the reviewers. Any product that may be evaluated in this article, or claim that may be made by its manufacturer, is not guaranteed or endorsed by the publisher.

## Supplementary Material

Supplementary Figure 1 Forest plot **(A)**, sensitivity analysis **(B)**, scatter plot **(C)**, and funnel plot **(D)** of the causal effect of *Eggerthella* on SLE risk.

Supplementary Figure 2 Forest plot **(A)**, sensitivity analysis **(B)**, scatter plot **(C)**, and funnel plot **(D)** of the causal effect of *Lactobacillales* on SLE risk.

Supplementary Figure 3 Forest plot **(A)**, sensitivity analysis **(B)**, scatter plot **(C)**, and funnel plot **(D)** of the causal effect of *Coprobacter* on SLE risk.

Supplementary Figure 4 Forest plot **(A)**, sensitivity analysis **(B)**, scatter plot **(C)**, and funnel plot **(D)** of the causal effect of *Bacillales* on SLE risk.

Supplementary Figure 5 Forest plot **(A)**, sensitivity analysis **(B)**, scatter plot **(C)**, and funnel plot **(D)** of the causal effect of *Lachnospira* on SLE risk.

Supplementary Figure 6 Forest plot **(A)**, sensitivity analysis **(B)**, scatter plot **(C)**, and funnel plot **(D)** of the causal effect of the whole gut microbiome on SLE risk (*P* < 5 × 10^-8^).
