# Supplementary figures and images for: Causal Effects of Gut Microbiome on Systemic Lupus Erythematosus: A Two-Sample Mendelian Randomization Study
Source: Front Immunol. 2021 Sep 7;12:667097. doi: 10.3389/fimmu.2021.667097 (PMC8453215; doi:10.3389/fimmu.2021.667097)

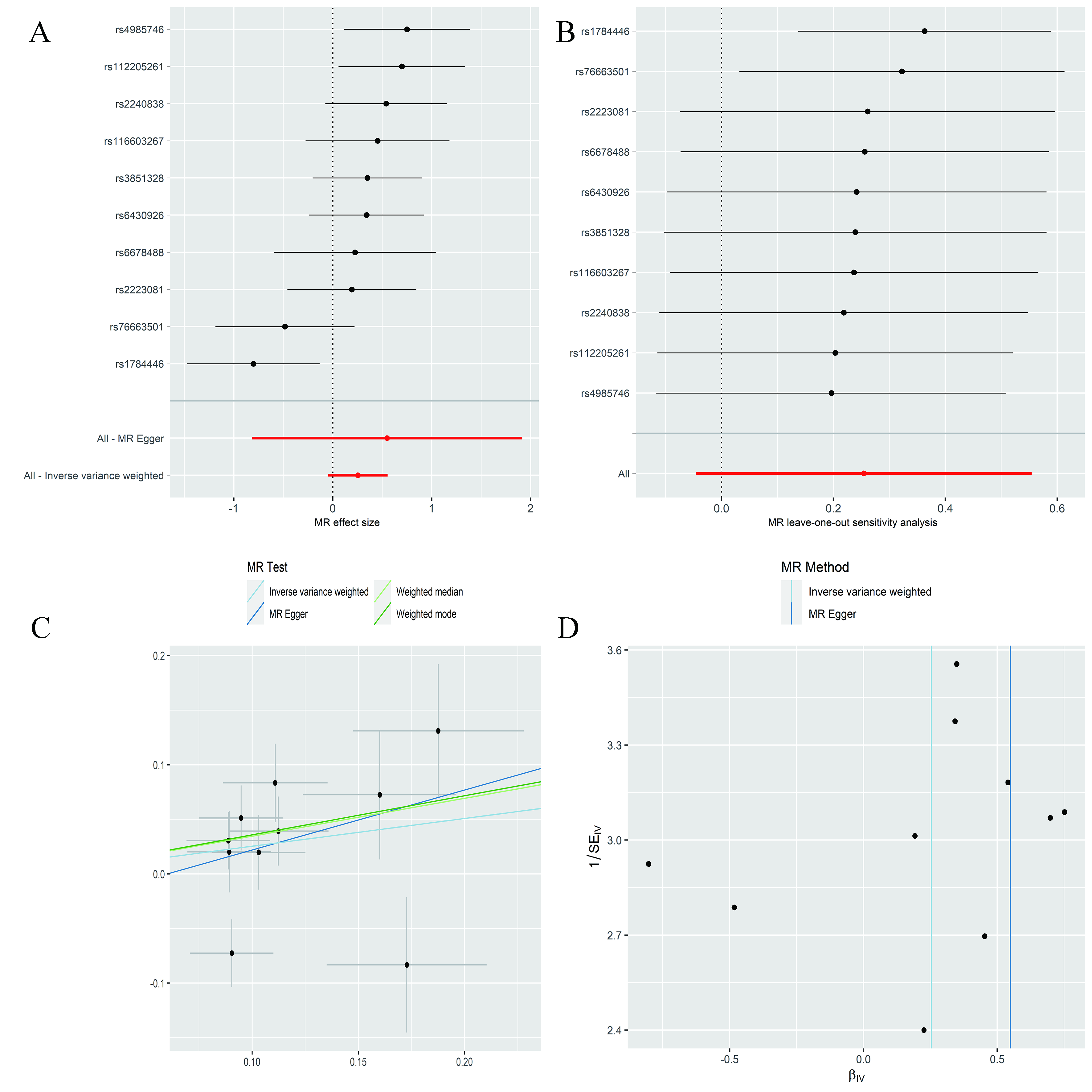

Supplement: Supplementary Figure 1 — Forest plot (A), sensitivity analysis (B), scatter plot (C), and funnel plot (D) of the causal effect of Eggerthella on SLE risk. [file Image_1.tif]

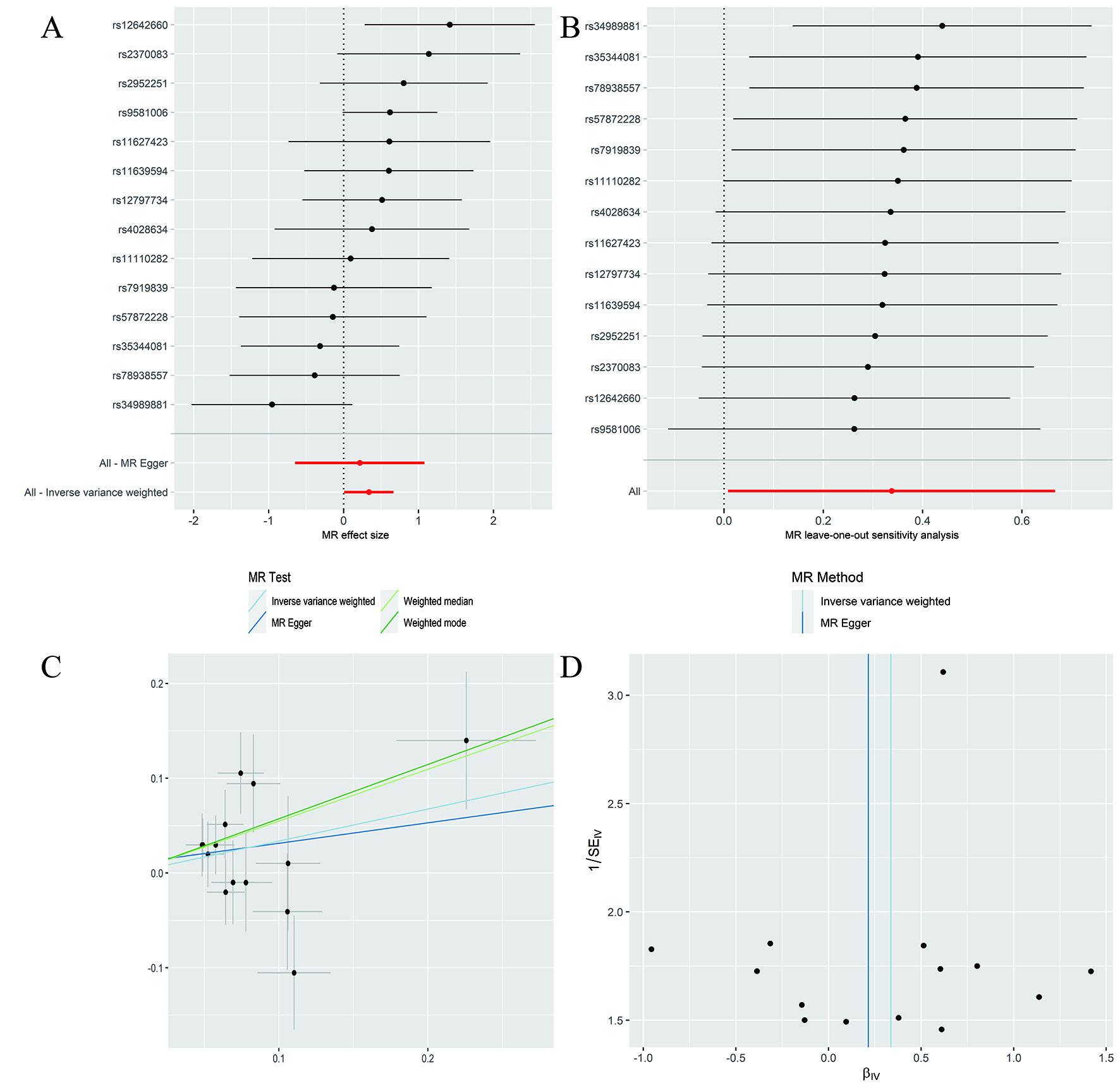

Supplement: Supplementary Figure 2 — Forest plot (A), sensitivity analysis (B), scatter plot (C), and funnel plot (D) of the causal effect of Lactobacillales on SLE risk. [file Image_2.tif]

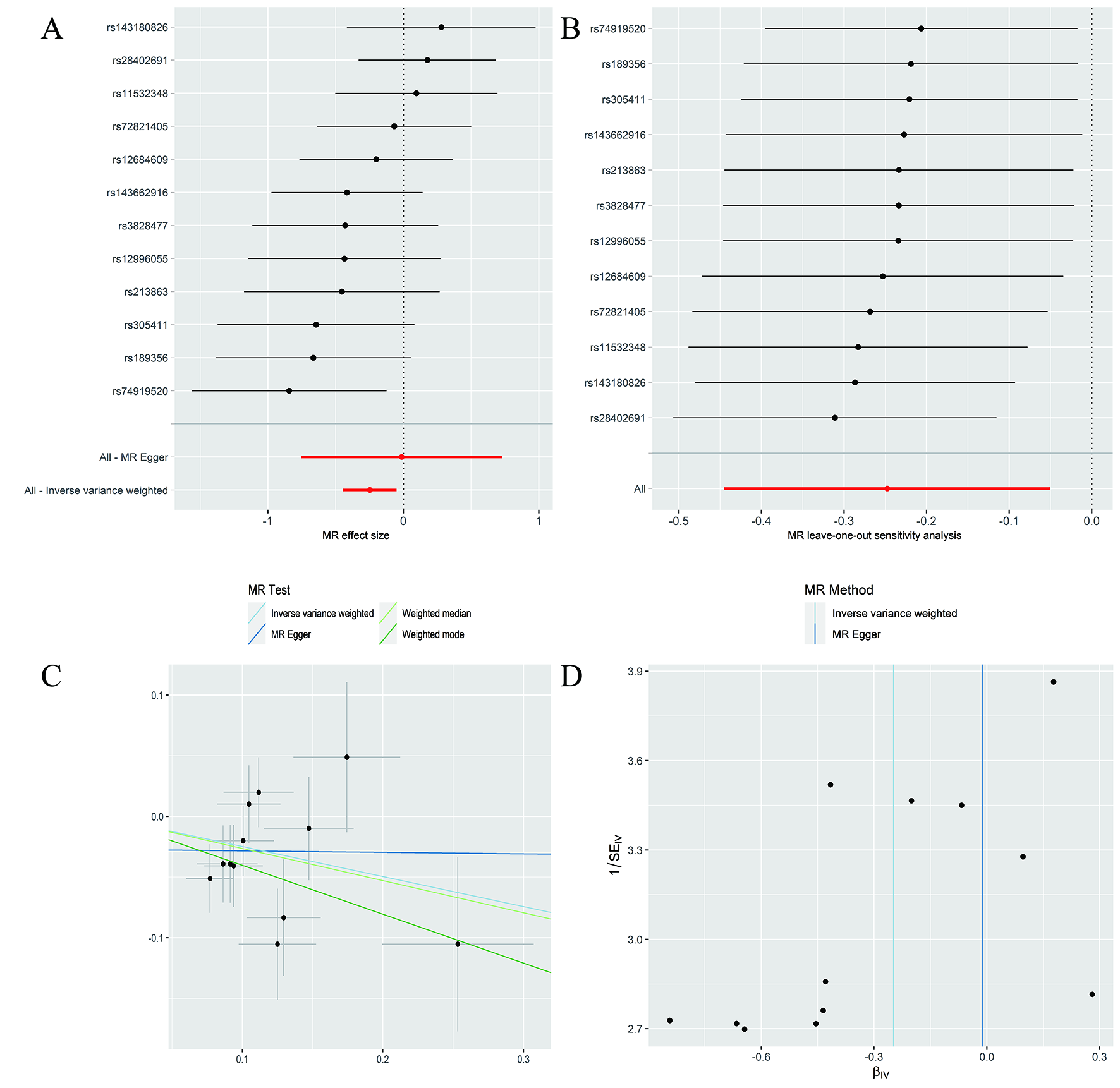

Supplement: Supplementary Figure 3 — Forest plot (A), sensitivity analysis (B), scatter plot (C), and funnel plot (D) of the causal effect of Coprobacter on SLE risk. [file Image_3.tif]

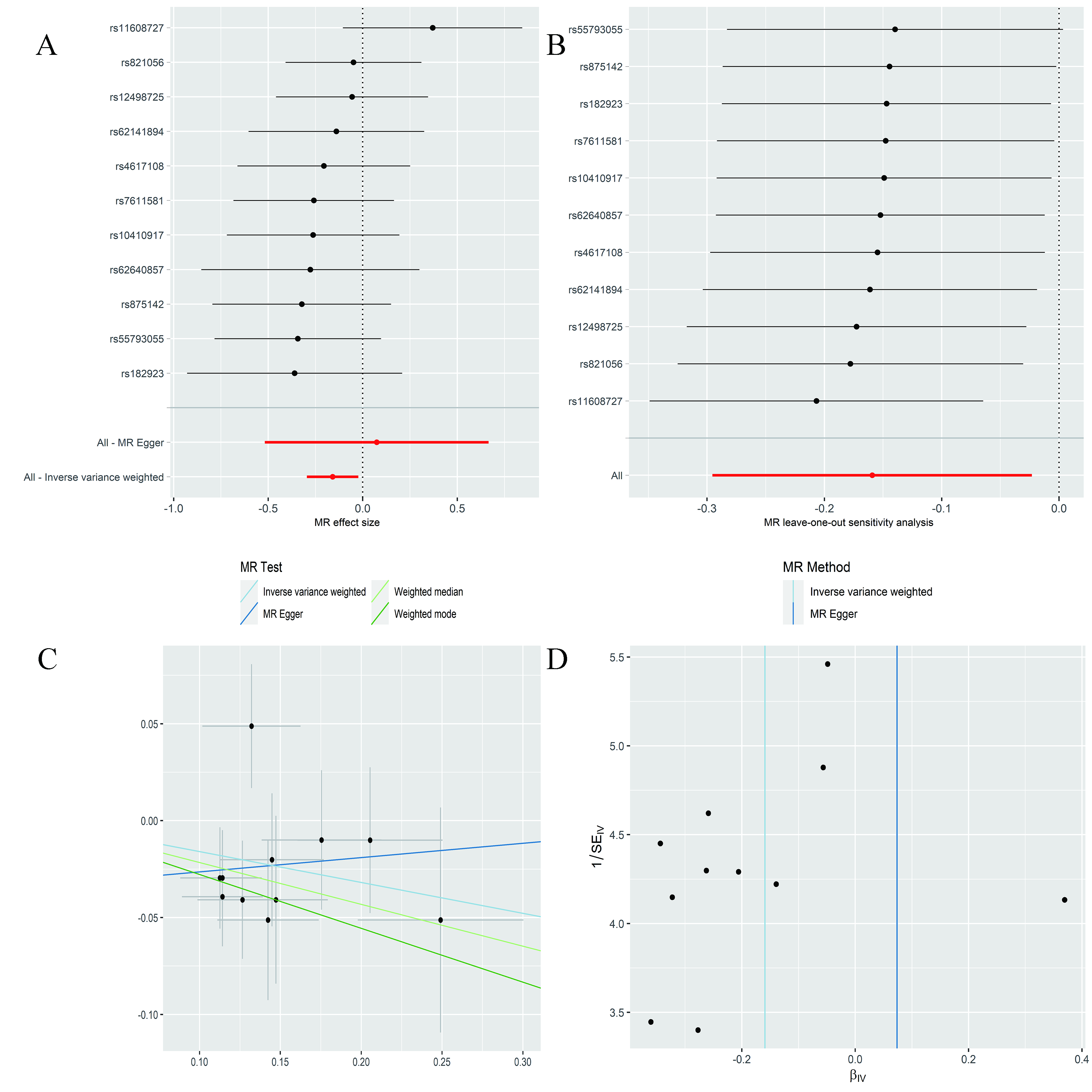

Supplement: Supplementary Figure 4 — Forest plot (A), sensitivity analysis (B), scatter plot (C), and funnel plot (D) of the causal effect of Bacillales on SLE risk. [file Image_4.tif]

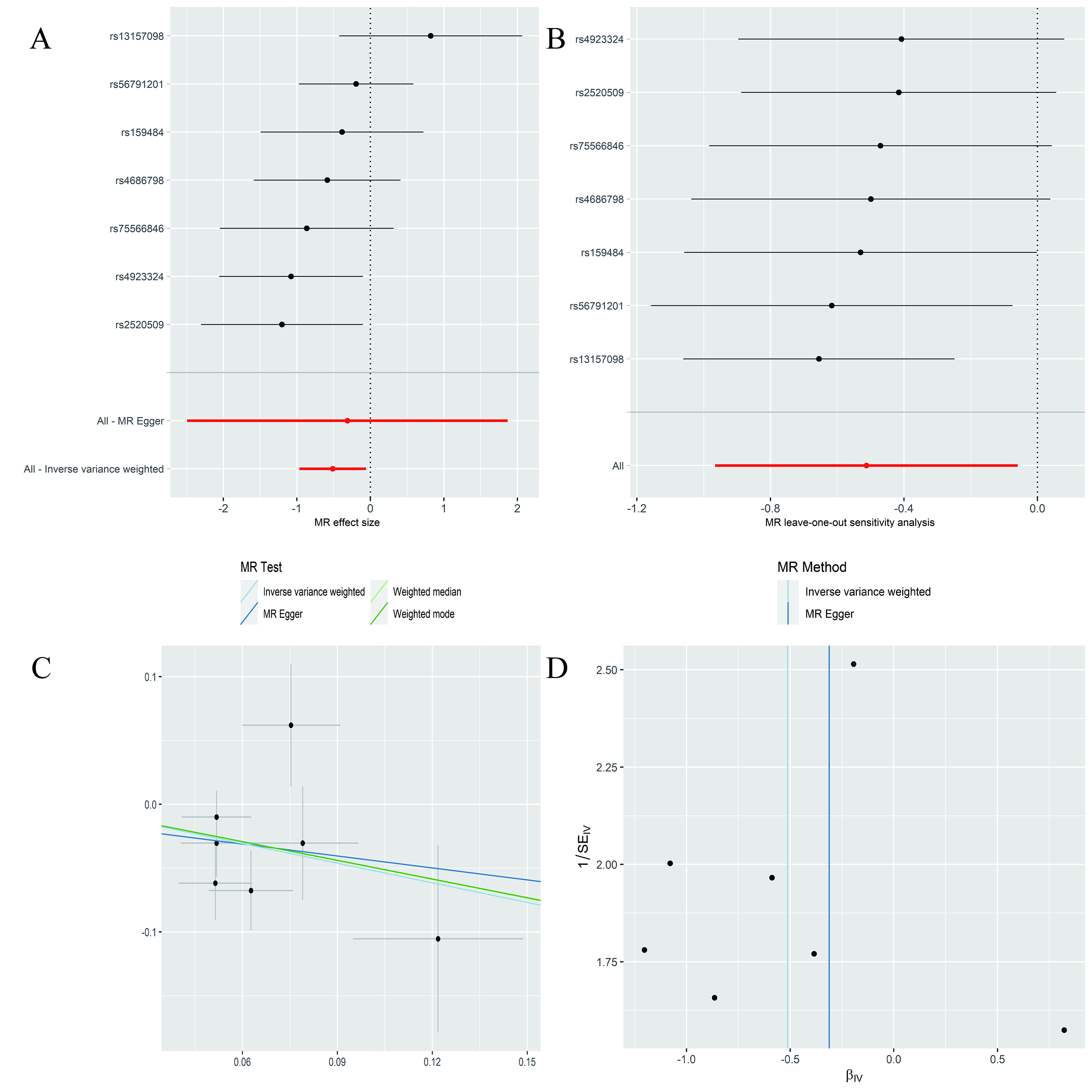

Supplement: Supplementary Figure 5 — Forest plot (A), sensitivity analysis (B), scatter plot (C), and funnel plot (D) of the causal effect of Lachnospira on SLE risk. [file Image_5.tif]

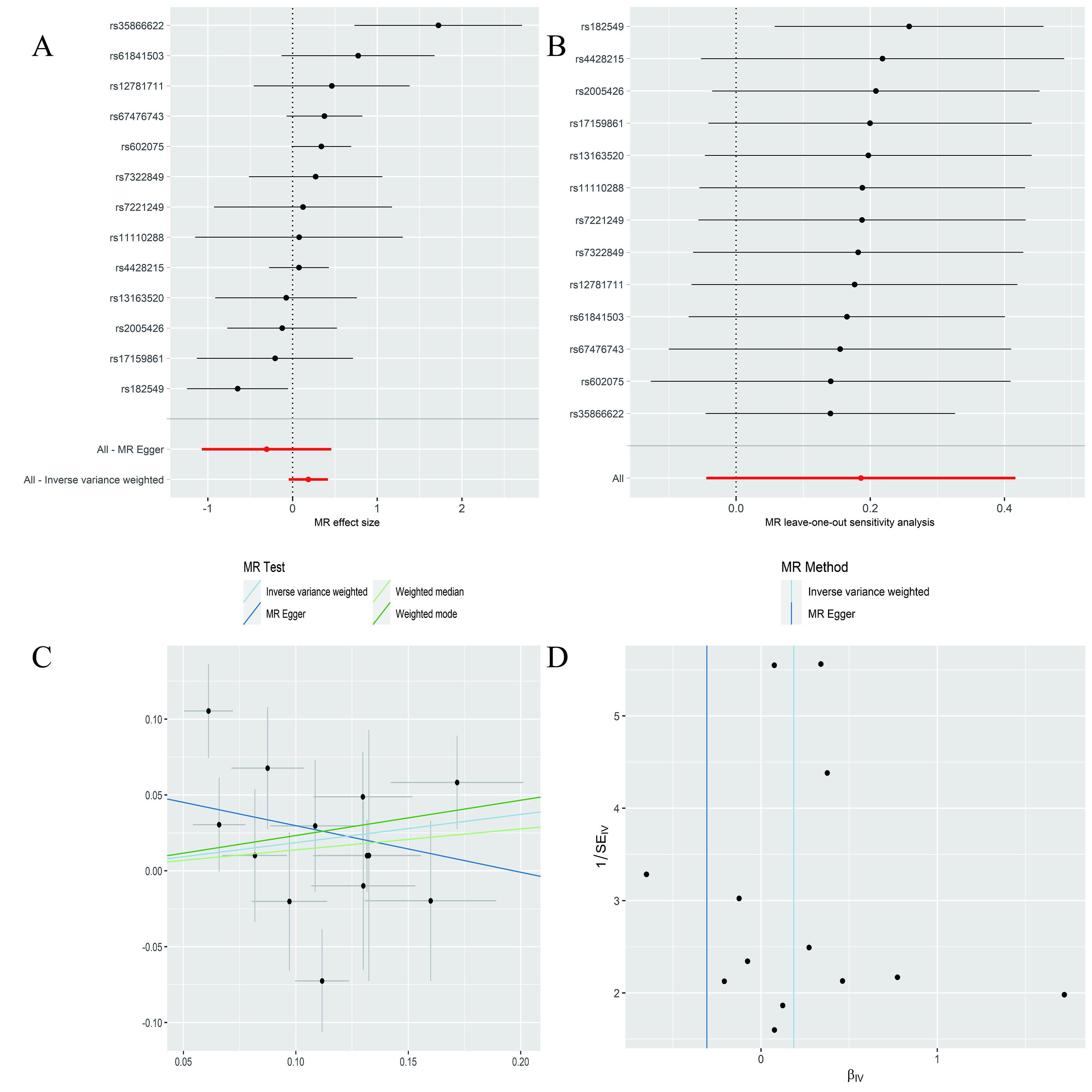

Supplement: Supplementary Figure 6 — Forest plot (A), sensitivity analysis (B), scatter plot (C), and funnel plot (D) of the causal effect of the whole gut microbiome on SLE risk (P < 5 × 10^-8^). [file Image_6.tif]
