# Supplementary material for: Causal Effects of Gut Microbiome on Systemic Lupus Erythematosus: A Two-Sample Mendelian Randomization Study
Source: Front Immunol. 2021 Sep 7;12:667097. doi: 10.3389/fimmu.2021.667097 (PMC8453215; doi:10.3389/fimmu.2021.667097)
Supplement: Supplementary file 7 [file Table_1.docx]

| **Supplementary Table 1. The estimates of two-sample MR of gut microbiome and SLE risk** | | | | | | | |
| --- | --- | --- | --- | --- | --- | --- | --- |
| **Classification** |  | **Nsnp** | **Methods** | **Beta** | **SE** | **OR (95% CI)** | ***P* value** |
| Phylum | Actinobacteria | 15 | MR Egger | -0.43 | 0.72 | 0.65 (0.16-2.67) | 0.560 |
|  |  |  | Weighted median | 0.00 | 0.21 | 1.00 (0.66-1.52) | 0.997 |
|  |  |  | Inverse variance weighted | -0.27 | 0.17 | 0.76 (0.55-1.07) | 0.115 |
|  |  |  | Weighted mode | 0.14 | 0.30 | 1.14 (0.63-2.07) | 0.661 |
|  | Bacteroidetes | 14 | MR Egger | 0.09 | 0.33 | 1.09 (0.57-2.09) | 0.794 |
|  |  |  | Weighted median | 0.31 | 0.20 | 1.37 (0.91-2.04) | 0.128 |
|  |  |  | Inverse variance weighted | 0.23 | 0.15 | 1.26 (0.95-1.69) | 0.111 |
|  |  |  | Weighted mode | 0.34 | 0.26 | 1.41 (0.85-2.33) | 0.201 |
|  | Cyanobacteria | 7 | MR Egger | 0.08 | 0.40 | 1.08 (0.50-2.34) | 0.857 |
|  |  |  | Weighted median | 0.25 | 0.17 | 1.29 (0.92-1.80) | 0.140 |
|  |  |  | Inverse variance weighted | 0.19 | 0.13 | 1.21 (0.94-1.56) | 0.136 |
|  |  |  | Weighted mode | 0.31 | 0.27 | 1.36 (0.81-2.29) | 0.292 |
|  | Euryarchaeota | 11 | MR Egger | 0.19 | 0.34 | 1.22 (0.62-2.39) | 0.585 |
|  |  |  | Weighted median | -0.07 | 0.12 | 0.94 (0.74-1.18) | 0.576 |
|  |  |  | Inverse variance weighted | -0.15 | 0.08 | 0.86 (0.73-1.01) | 0.071 |
|  |  |  | Weighted mode | -0.07 | 0.14 | 0.93 (0.71-1.22) | 0.623 |
|  | Firmicutes | 15 | MR Egger | 0.28 | 0.32 | 1.32 (0.71-2.45) | 0.397 |
|  |  |  | Weighted median | 0.17 | 0.18 | 1.12 (0.83-1.69) | 0.343 |
|  |  |  | Inverse variance weighted | 0.05 | 0.14 | 1.05 (0.79-1.39) | 0.729 |
|  |  |  | Weighted mode | 0.23 | 0.27 | 1.26 (0.75-2.12) | 0.394 |
|  | Lentisphaerae | 9 | MR Egger | 0.66 | 0.38 | 1.93 (0.92-4.08) | 0.127 |
|  |  |  | Weighted median | 0.13 | 0.14 | 1.14 (0.86-1.51) | 0.364 |
|  |  |  | Inverse variance weighted | 0.08 | 0.10 | 1.08 (0.88-1.32) | 0.455 |
|  |  |  | Weighted mode | 0.19 | 0.22 | 1.22 (0.79-1.86) | 0.396 |
|  | Proteobacteria | 9 | MR Egger | -0.66 | 0.73 | 0.52 (0.12-2.15) | 0.394 |
|  |  |  | Weighted median | 0.18 | 0.29 | 1.20 (0.68-2.12) | 0.535 |
|  |  |  | Inverse variance weighted | -0.01 | 0.25 | 0.99 (0.61-1.60) | 0.954 |
|  |  |  | Weighted mode | 0.43 | 0.55 | 1.54 (0.52-4.55) | 0.454 |
|  | Tenericutes | 11 | MR Egger | -0.25 | 0.50 | 0.78 (0.29-2.07) | 0.632 |
|  |  |  | Weighted median | -0.11 | 0.20 | 0.90 (0.61-1.33) | 0.592 |
|  |  |  | Inverse variance weighted | -0.13 | 0.15 | 0.88 (0.66-1.17) | 0.375 |
|  |  |  | Weighted mode | 0.03 | 0.31 | 1.03 (0.55-1.90) | 0.937 |
|  | Verrucomicrobia | 12 | MR Egger | 0.49 | 0.45 | 1.64 (0.68-3.94) | 0.294 |
|  |  |  | Weighted median | 0.14 | 0.19 | 1.15 (0.80-1.66) | 0.441 |
|  |  |  | Inverse variance weighted | 0.14 | 0.16 | 1.15 (0.85-1.57) | 0.359 |
|  |  |  | Weighted mode | 0.11 | 0.28 | 1.11 (0.65-1.91) | 0.705 |
| Class | Actinobacteria | 20 | MR Egger | -0.32 | 0.39 | 0.73 (0.34-1.57) | 0.428 |
|  |  |  | Weighted median | -0.16 | 0.16 | 0.85 (0.62-1.16) | 0.310 |
|  |  |  | Inverse variance weighted | -0.19 | 0.11 | 0.83 (0.66-1.04) | 0.101 |
|  |  |  | Weighted mode | -0.19 | 0.26 | 0.83 (0.49-1.39) | 0.486 |
|  | Alphaproteobacteria | 7 | MR Egger | -0.63 | 0.80 | 0.53 (0.11-2.56) | 0.468 |
|  |  |  | Weighted median | -0.31 | 0.23 | 0.73 (0.47-1.15) | 0.172 |
|  |  |  | Inverse variance weighted | -0.09 | 0.23 | 0.91 (0.58-1.42) | 0.677 |
|  |  |  | Weighted mode | -0.35 | 0.29 | 0.71 (0.40-1.24) | 0.275 |
|  | Bacilli | 16 | MR Egger | 0.61 | 0.44 | 1.84 (0.77-4.38) | 0.189 |
|  |  |  | Weighted median | 0.46 | 0.21 | 1.59 (1.06-2.39) | 0.027 |
|  |  |  | Inverse variance weighted | 0.34 | 0.16 | 1.40 (1.02-1.93) | 0.037 |
|  |  |  | Weighted mode | 0.65 | 0.34 | 1.91 (0.98-3.75) | 0.078 |
|  | Bacteroidia | 14 | MR Egger | -0.43 | 0.31 | 0.65 (0.36-1.19) | 0.190 |
|  |  |  | Weighted median | 0.24 | 0.20 | 1.27 (0.86-1.89) | 0.235 |
|  |  |  | Inverse variance weighted | 0.11 | 0.15 | 1.11 (0.84-1.48) | 0.456 |
|  |  |  | Weighted mode | 0.28 | 0.29 | 1.32 (0.75-2.35) | 0.355 |
|  | Betaproteobacteria | 13 | MR Egger | -0.07 | 0.32 | 0.93 (0.50-1.75) | 0.833 |
|  |  |  | Weighted median | 0.01 | 0.18 | 1.01 (0.70-1.44) | 0.977 |
|  |  |  | Inverse variance weighted | -0.07 | 0.13 | 0.94 (0.72-1.22) | 0.623 |
|  |  |  | Weighted mode | 0.07 | 0.21 | 1.07 (0.71-1.61) | 0.762 |
|  | Clostridia | 13 | MR Egger | 0.57 | 0.32 | 1.77 (0.95-3.32) | 0.101 |
|  |  |  | Weighted median | -0.14 | 0.21 | 0.87 (0.58-1.30) | 0.493 |
|  |  |  | Inverse variance weighted | -0.23 | 0.15 | 0.80 (0.59-1.08) | 0.138 |
|  |  |  | Weighted mode | 0.29 | 0.28 | 1.34 (0.77-2.32) | 0.316 |
|  | Coriobacteriia | 14 | MR Egger | -0.44 | 1.06 | 0.64 (0.08- 5.15) | 0.686 |
|  |  |  | Weighted median | -0.23 | 0.26 | 0.79 (0.48-1.31) | 0.371 |
|  |  |  | Inverse variance weighted | -0.06 | 0.23 | 0.94 (0.60-1.49) | 0.234 |
|  |  |  | Weighted mode | -0.67 | 0.47 | 0.51 (0.20-1.29) | 0.181 |
|  | Deltaproteobacteria | 11 | MR Egger | 1.53 | 0.50 | 4.62 (1.73- 12.35) | 0.014 |
|  |  |  | Weighted median | 0.17 | 0.26 | 1.18 (0.71-1.95) | 0.515 |
|  |  |  | Inverse variance weighted | 0.31 | 0.24 | 1.36 (0.85-2.18) | 0.202 |
|  |  |  | Weighted mode | -0.42 | 0.56 | 0.66 (0.22-1.95) | 0.467 |
|  | Erysipelotrichia | 13 | MR Egger | -1.23 | 1.27 | 0.29 (0.02- 3.49) | 0.351 |
|  |  |  | Weighted median | 0.14 | 0.27 | 1.15 (0.68-1.95) | 0.608 |
|  |  |  | Inverse variance weighted | -0.07 | 0.26 | 0.94 (0.57-1.55) | 0.798 |
|  |  |  | Weighted mode | 0.68 | 0.51 | 1.98 (0.73-5.41) | 0.206 |
|  | Gammaproteobacteria | 5 | MR Egger | -1.10 | 0.70 | 0.33 (0.08-1.30) | 0.212 |
|  |  |  | Weighted median | 0.07 | 0.29 | 1.07 (0.61-1.89) | 0.808 |
|  |  |  | Inverse variance weighted | -0.04 | 0.25 | 0.96 (0.59-1.57) | 0.876 |
|  |  |  | Weighted mode | 0.41 | 0.47 | 1.51 (0.60-3.82) | 0.431 |
|  | Lentisphaeria | 7 | MR Egger | 0.23 | 0.54 | 1.26 (0.43-3.64) | 0.693 |
|  |  |  | Weighted median | -0.02 | 0.17 | 0.98 (0.70-1.37) | 0.904 |
|  |  |  | Inverse variance weighted | 0.00 | 0.15 | 1.00 (0.74-1.35) | 0.977 |
|  |  |  | Weighted mode | -0.07 | 0.26 | 0.93 (0.55-1.55) | 0.786 |
|  | Melainabacteria | 8 | MR Egger | -0.17 | 0.37 | 0.84 (0.40-1.75) | 0.659 |
|  |  |  | Weighted median | -0.10 | 0.15 | 0.91 (0.68-1.21) | 0.517 |
|  |  |  | Inverse variance weighted | -0.03 | 0.11 | 0.97 (0.78-1.20) | 0.776 |
|  |  |  | Weighted mode | -0.12 | 0.18 | 0.89 (0.62-1.27) | 0.529 |
|  | Methanobacteria | 8 | MR Egger | 0.44 | 0.48 | 1.55 (0.61-3.94) | 0.391 |
|  |  |  | Weighted median | -0.05 | 0.12 | 0.95 (0.75-1.21) | 0.680 |
|  |  |  | Inverse variance weighted | -0.03 | 0.13 | 0.97 (0.76-1.24) | 0.810 |
|  |  |  | Weighted mode | -0.04 | 0.15 | 0.96 (0.72-1.29) | 0.800 |
|  | Mollicutes | 11 | MR Egger | -0.25 | 0.50 | 0.78 (0.29-2.07) | 0.632 |
|  |  |  | Weighted median | -0.11 | 0.20 | 0.90 (0.60-1.34) | 0.601 |
|  |  |  | Inverse variance weighted | -0.13 | 0.15 | 0.88 (0.66-1.17) | 0.375 |
|  |  |  | Weighted mode | 0.03 | 0.29 | 1.03 (0.58-1.81) | 0.932 |
|  | Negativicutes | 9 | MR Egger | 0.82 | 1.18 | 2.28 (0.22-23.17) | 0.508 |
|  |  |  | Weighted median | 0.05 | 0.30 | 1.05 (0.58-1.91) | 0.860 |
|  |  |  | Inverse variance weighted | -0.06 | 0.28 | 0.95 (0.55-1.64) | 0.842 |
|  |  |  | Weighted mode | 0.06 | 0.46 | 1.06 (0.43-2.61) | 0.897 |
|  | Verrucomicrobiae | 11 | MR Egger | 0.64 | 0.62 | 1.90 (0.57-6.38) | 0.324 |
|  |  |  | Weighted median | -0.13 | 0.21 | 0.88 (0.58-1.32) | 0.525 |
|  |  |  | Inverse variance weighted | 0.10 | 0.18 | 1.10 (0.78-1.56) | 0.578 |
|  |  |  | Weighted mode | -0.23 | 0.38 | 0.79 (0.37-1.68) | 0.558 |
| Order | Actinomycetales | 5 | MR Egger | 0.32 | 0.76 | 1.37 (0.31-6.11) | 0.705 |
|  |  |  | Weighted median | 0.10 | 0.23 | 1.11 (0.70-1.75) | 0.656 |
|  |  |  | Inverse variance weighted | 0.00 | 0.27 | 1.00 (0.59-1.68) | 0.986 |
|  |  |  | Weighted mode | 0.11 | 0.26 | 1.11 (0.67-1.85) | 0.701 |
|  | Bacillales | 11 | MR Egger | 0.07 | 0.30 | 1.08 (0.60-1.95) | 0.813 |
|  |  |  | Weighted median | -0.22 | 0.09 | 0.81 (0.67-0.96) | 0.018 |
|  |  |  | Inverse variance weighted | -0.16 | 0.07 | 0.85 (0.74-0.98) | 0.022 |
|  |  |  | Weighted mode | -0.28 | 0.14 | 0.76 (0.57-1.01) | 0.080 |
|  | Bacteroidales | 14 | MR Egger | -0.43 | 0.31 | 0.65 (0.36-1.19) | 0.190 |
|  |  |  | Weighted median | 0.24 | 0.21 | 1.27 (0.85-1.91) | 0.245 |
|  |  |  | Inverse variance weighted | 0.11 | 0.15 | 1.11 (0.84-1.48) | 0.456 |
|  |  |  | Weighted mode | 0.28 | 0.27 | 1.32 (0.77-2.27) | 0.326 |
|  | Bifidobacteriales | 11 | MR Egger | -0.61 | 0.46 | 0.54 (0.22-1.35) | 0.223 |
|  |  |  | Weighted median | 0.18 | 0.20 | 1.19 (0.81-1.75) | 0.371 |
|  |  |  | Inverse variance weighted | 0.02 | 0.14 | 1.02 (0.78-1.35) | 0.864 |
|  |  |  | Weighted mode | 0.23 | 0.36 | 1.26 (0.62-2.56) | 0.545 |
|  | Burkholderiales | 13 | MR Egger | 0.32 | 0.35 | 1.38 (0.69-2.77) | 0.382 |
|  |  |  | Weighted median | 0.06 | 0.19 | 1.06 (0.73-1.53) | 0.757 |
|  |  |  | Inverse variance weighted | 0.10 | 0.16 | 1.11 (0.82-1.50) | 0.511 |
|  |  |  | Weighted mode | 0.04 | 0.22 | 1.04 (0.68-1.60) | 0.855 |
|  | Clostridiales | 11 | MR Egger | 0.58 | 0.32 | 1.78 (0.95-3.34) | 0.105 |
|  |  |  | Weighted median | -0.12 | 0.22 | 0.88 (0.58-1.35) | 0.564 |
|  |  |  | Inverse variance weighted | -0.18 | 0.16 | 0.84 (0.61-1.15) | 0.274 |
|  |  |  | Weighted mode | 0.28 | 0.25 | 1.33 (0.81-2.18) | 0.288 |
|  | Coriobacteriales | 14 | MR Egger | -0.44 | 1.06 | 0.64 (0.08- 5.15) | 0.686 |
|  |  |  | Weighted median | -0.23 | 0.27 | 0.79 (0.47-1.34) | 0.390 |
|  |  |  | Inverse variance weighted | -0.06 | 0.23 | 0.94 (0.60-1.49) | 0.806 |
|  |  |  | Weighted mode | -0.67 | 0.44 | 0.51 (0.21-1.22) | 0.155 |
|  | Desulfovibrionales | 11 | MR Egger | 1.57 | 0.46 | 4.81 (1.94-11.88) | 0.008 |
|  |  |  | Weighted median | 0.11 | 0.25 | 1.12 (0.69-1.82) | 0.647 |
|  |  |  | Inverse variance weighted | 0.25 | 0.24 | 1.28 (0.80-2.05) | 0.304 |
|  |  |  | Weighted mode | -0.40 | 0.58 | 0.67 (0.22-2.07) | 0.500 |
|  | Enterobacteriales | 8 | MR Egger | 0.40 | 1.07 | 1.50 (0.18-12.25) | 0.720 |
|  |  |  | Weighted median | -0.35 | 0.23 | 0.70 (0.45-1.10) | 0.124 |
|  |  |  | Inverse variance weighted | -0.19 | 0.18 | 0.83 (0.58-1.17) | 0.283 |
|  |  |  | Weighted mode | -0.42 | 0.38 | 0.65 (0.31-1.37) | 0.297 |
|  | Erysipelotrichales | 13 | MR Egger | -1.23 | 1.27 | 0.29 (0.02-3.49) | 0.351 |
|  |  |  | Weighted median | 0.14 | 0.26 | 1.15 (0.70-1.89) | 0.586 |
|  |  |  | Inverse variance weighted | -0.07 | 0.26 | 0.94 (0.57-1.55) | 0.798 |
|  |  |  | Weighted mode | 0.68 | 0.51 | 1.98 (0.73- 5.41) | 0.206 |
|  | Gastranaerophilales | 7 | MR Egger | -0.05 | 0.48 | 0.95 (0.37-2.44) | 0.924 |
|  |  |  | Weighted median | -0.08 | 0.16 | 0.92 (0.68-1.25) | 0.595 |
|  |  |  | Inverse variance weighted | -0.01 | 0.12 | 0.99 (0.79-1.25) | 0.945 |
|  |  |  | Weighted mode | -0.11 | 0.18 | 0.90 (0.62-1.29) | 0.573 |
|  | Lactobacillales | 14 | MR Egger | 0.22 | 0.44 | 1.24 (0.52-2.94) | 0.634 |
|  |  |  | Weighted median | 0.55 | 0.22 | 1.73 (1.13-2.64) | 0.011 |
|  |  |  | Inverse variance weighted | 0.34 | 0.17 | 1.40 (1.01-1.95) | 0.045 |
|  |  |  | Weighted mode | 0.57 | 0.33 | 1.78 (0.93-3.34) | 0.107 |
|  | Methanobacteriales | 8 | MR Egger | 0.44 | 0.48 | 1.55 (0.61-3.94) | 0.391 |
|  |  |  | Weighted median | -0.05 | 0.12 | 0.95 (0.75-1.20) | 0.673 |
|  |  |  | Inverse variance weighted | -0.03 | 0.13 | 0.97 (0.76-1.24) | 0.810 |
|  |  |  | Weighted mode | -0.04 | 0.15 | 0.96 (0.72-1.28) | 0.797 |
|  | MollicutesRF9 | 12 | MR Egger | -0.29 | 0.43 | 0.75 (0.32-1.73) | 0.511 |
|  |  |  | Weighted median | -0.06 | 0.18 | 0.94 (0.66-1.35) | 0.733 |
|  |  |  | Inverse variance weighted | -0.05 | 0.13 | 0.95 (0.73-1.23) | 0.691 |
|  |  |  | Weighted mode | 0.05 | 0.29 | 1.05 (0.59-1.85) | 0.874 |
|  | NB1n | 12 | MR Egger | 0.52 | 0.46 | 1.68 (0.69-4.10) | 0.283 |
|  |  |  | Weighted median | 0.09 | 0.14 | 1.09 (0.84-1.43) | 0.508 |
|  |  |  | Inverse variance weighted | 0.04 | 0.11 | 1.04 (0.83-1.30) | 0.743 |
|  |  |  | Weighted mode | 0.14 | 0.23 | 1.16 (0.73-1.82) | 0.545 |
|  | Pasteurellales | 14 | MR Egger | -0.09 | 0.24 | 0.92 (0.58-1.45) | 0.716 |
|  |  |  | Weighted median | -0.11 | 0.13 | 0.90 (0.70-1.16) | 0.408 |
|  |  |  | Inverse variance weighted | -0.16 | 0.10 | 0.86 (0.71-1.03) | 0.104 |
|  |  |  | Weighted mode | -0.11 | 0.19 | 0.90 (0.62-1.29) | 0.564 |
|  | Rhodospirillales | 12 | MR Egger | -0.26 | 0.86 | 0.77 (0.14-4.13) | 0.768 |
|  |  |  | Weighted median | -0.14 | 0.18 | 0.87 (0.61-1.25) | 0.449 |
|  |  |  | Inverse variance weighted | -0.06 | 0.16 | 0.94 (0.69-1.28) | 0.696 |
|  |  |  | Weighted mode | -0.37 | 0.33 | 0.69 (0.36-1.32) | 0.286 |
|  | Selenomonadales | 9 | MR Egger | 0.82 | 1.18 | 2.28 (0.22-23.17) | 0.508 |
|  |  |  | Weighted median | 0.05 | 0.31 | 1.05 (0.57-1.94) | 0.864 |
|  |  |  | Inverse variance weighted | -0.06 | 0.28 | 0.95 (0.55-1.64) | 0.842 |
|  |  |  | Weighted mode | 0.06 | 0.45 | 1.06 (0.44-2.56) | 0.895 |
|  | Verrucomicrobiales | 11 | MR Egger | 0.64 | 0.62 | 1.90 (0.57-6.38) | 0.324 |
|  |  |  | Weighted median | -0.13 | 0.21 | 0.88 (0.58-1.33) | 0.535 |
|  |  |  | Inverse variance weighted | 0.10 | 0.18 | 1.10 (0.78-1.56) | 0.578 |
|  |  |  | Weighted mode | -0.23 | 0.37 | 0.79 (0.38-1.64) | 0.544 |
|  | Victivallales | 7 | MR Egger | 0.23 | 0.54 | 1.26 (0.43-3.64) | 0.693 |
|  |  |  | Weighted median | -0.02 | 0.17 | 0.98 (0.70-1.36) | 0.903 |
|  |  |  | Inverse variance weighted | 0.00 | 0.15 | 1.00 (0.74-1.35) | 0.977 |
|  |  |  | Weighted mode | -0.07 | 0.27 | 0.93 (0.55-1.57) | 0.791 |
| Family | Acidaminococcaceae | 6 | MR Egger | -0.58 | 0.59 | 0.56 (0.18-1.78) | 0.383 |
|  |  |  | Weighted median | 0.09 | 0.24 | 1.09 (0.68-1.76) | 0.720 |
|  |  |  | Inverse variance weighted | 0.09 | 0.19 | 1.09 (0.75-1.58) | 0.654 |
|  |  |  | Weighted mode | 0.09 | 0.31 | 1.10 (0.60-2.02) | 0.776 |
|  | Actinomycetaceae | 5 | MR Egger | 0.32 | 0.76 | 1.38 (0.31-6.11) | 0.700 |
|  |  |  | Weighted median | 0.10 | 0.24 | 1.11 (0.70-1.77) | 0.664 |
|  |  |  | Inverse variance weighted | 0.00 | 0.27 | 1.00 (0.59-1.68) | 0.989 |
|  |  |  | Weighted mode | 0.11 | 0.26 | 1.12 (0.67-1.88) | 0.694 |
|  | Alcaligenaceae | 12 | MR Egger | 0.04 | 0.77 | 1.04 (0.23-4.73) | 0.960 |
|  |  |  | Weighted median | -0.13 | 0.21 | 0.88 (0.57-1.33) | 0.538 |
|  |  |  | Inverse variance weighted | 0.11 | 0.16 | 1.11 (0.81-1.52) | 0.506 |
|  |  |  | Weighted mode | -0.18 | 0.29 | 0.83 (0.47-1.48) | 0.546 |
|  | Bacteroidaceae | 12 | MR Egger | -0.44 | 1.07 | 0.65 (0.08-5.24) | 0.691 |
|  |  |  | Weighted median | -0.21 | 0.26 | 0.81 (0.49-1.34) | 0.412 |
|  |  |  | Inverse variance weighted | -0.02 | 0.20 | 0.98 (0.66-1.45) | 0.902 |
|  |  |  | Weighted mode | -0.46 | 0.48 | 0.63 (0.25-1.60) | 0.351 |
|  | BacteroidalesS24.7 | 4 | MR Egger | 0.61 | 0.77 | 1.84 (0.41-8.37) | 0.511 |
|  |  |  | Weighted median | 0.21 | 0.27 | 1.23 (0.72-2.11) | 0.447 |
|  |  |  | Inverse variance weighted | 0.15 | 0.23 | 1.17 (0.74-1.83) | 0.502 |
|  |  |  | Weighted mode | 0.23 | 0.29 | 1.26 (0.71-2.25) | 0.483 |
|  | Bifidobacteriaceae | 11 | MR Egger | -0.61 | 0.46 | 0.54 (0.22-1.35) | 0.223 |
|  |  |  | Weighted median | 0.18 | 0.20 | 1.19 (0.81-1.75) | 0.372 |
|  |  |  | Inverse variance weighted | 0.02 | 0.14 | 1.02 (0.78-1.35) | 0.864 |
|  |  |  | Weighted mode | 0.23 | 0.36 | 1.26 (0.61-2.56) | 0.547 |
|  | Christensenellaceae | 13 | MR Egger | -0.46 | 0.32 | 0.63 (0.34-1.18) | 0.176 |
|  |  |  | Weighted median | 0.23 | 0.21 | 1.26 (0.84-1.89) | 0.266 |
|  |  |  | Inverse variance weighted | 0.09 | 0.15 | 1.09 (0.82-1.46) | 0.561 |
|  |  |  | Weighted mode | 0.27 | 0.32 | 1.31 (0.70-2.47) | 0.414 |
|  | Clostridiaceae1 | 7 | MR Egger | -0.08 | 0.50 | 0.92 (0.35-2.47) | 0.881 |
|  |  |  | Weighted median | -0.09 | 0.21 | 0.91 (0.60-1.39) | 0.671 |
|  |  |  | Inverse variance weighted | -0.25 | 0.17 | 0.78 (0.56-1.08) | 0.131 |
|  |  |  | Weighted mode | -0.07 | 0.25 | 0.93 (0.57-1.53) | 0.785 |
|  | ClostridialesvadinBB60 | 15 | MR Egger | -0.05 | 0.35 | 0.95 (0.48-1.87) | 0.880 |
|  |  |  | Weighted median | 0.13 | 0.17 | 1.14 (0.82-1.59) | 0.440 |
|  |  |  | Inverse variance weighted | 0.06 | 0.12 | 1.06 (0.83-1.35) | 0.636 |
|  |  |  | Weighted mode | -0.36 | 0.31 | 0.70 (0.38-1.29) | 0.273 |
|  | Coriobacteriaceae | 14 | MR Egger | -0.44 | 1.06 | 0.64 (0.08-5.15) | 0.686 |
|  |  |  | Weighted median | -0.23 | 0.25 | 0.79 (0.48-1.31) | 0.364 |
|  |  |  | Inverse variance weighted | -0.06 | 0.23 | 0.94 (0.60-1.49) | 0.806 |
|  |  |  | Weighted mode | -0.67 | 0.45 | 0.51 (0.21-1.24) | 0.163 |
|  | Defluviitaleaceae | 10 | MR Egger | 0.20 | 0.42 | 1.23 (0.54-2.78) | 0.637 |
|  |  |  | Weighted median | 0.12 | 0.16 | 1.12 (0.82-1.54) | 0.466 |
|  |  |  | Inverse variance weighted | 0.08 | 0.12 | 1.08 (0.86-1.37) | 0.508 |
|  |  |  | Weighted mode | -0.08 | 0.24 | 0.92 (0.57-1.48) | 0.740 |
|  | Desulfovibrionaceae | 11 | MR Egger | 1.46 | 0.48 | 4.31 (1.67-11.10) | 0.014 |
|  |  |  | Weighted median | 0.24 | 0.24 | 1.27 (0.79-2.05) | 0.330 |
|  |  |  | Inverse variance weighted | 0.27 | 0.23 | 1.30 (0.83-2.06) | 0.254 |
|  |  |  | Weighted mode | -0.38 | 0.54 | 0.68 (0.24-1.96) | 0.491 |
|  | Enterobacteriaceae | 8 | MR Egger | 0.40 | 1.07 | 1.50 (0.18-12.25) | 0.720 |
|  |  |  | Weighted median | -0.35 | 0.23 | 0.70 (0.45-1.10) | 0.120 |
|  |  |  | Inverse variance weighted | -0.19 | 0.18 | 0.83 (0.58-1.17) | 0.283 |
|  |  |  | Weighted mode | -0.42 | 0.33 | 0.65 (0.34-1.26) | 0.246 |
|  | Erysipelotrichaceae | 13 | MR Egger | -1.23 | 1.27 | 0.29 (0.02-3.49) | 0.351 |
|  |  |  | Weighted median | 0.14 | 0.25 | 1.15 (0.70-1.87) | 0.578 |
|  |  |  | Inverse variance weighted | -0.07 | 0.26 | 0.94 (0.57-1.55) | 0.798 |
|  |  |  | Weighted mode | 0.68 | 0.54 | 1.98 (0.69-5.66) | 0.225 |
|  | FamilyXI (ID: 1936) | 11 | MR Egger | -0.10 | 0.54 | 0.91 (0.31-2.61) | 0.860 |
|  |  |  | Weighted median | -0.06 | 0.11 | 0.94 (0.75-1.17) | 0.573 |
|  |  |  | Inverse variance weighted | 0.01 | 0.09 | 1.01 (0.85-1.21) | 0.868 |
|  |  |  | Weighted mode | -0.09 | 0.15 | 0.91 (0.68-1.23) | 0.560 |
|  | FamilyXIII (ID: 1957) | 14 | MR Egger | -0.47 | 0.65 | 0.62 (0.17-2.25) | 0.486 |
|  |  |  | Weighted median | -0.37 | 0.22 | 0.69 (0.45-1.06) | 0.086 |
|  |  |  | Inverse variance weighted | -0.16 | 0.16 | 0.85 (0.62-1.16) | 0.306 |
|  |  |  | Weighted mode | -0.47 | 0.35 | 0.62 (0.32-1.23) | 0.196 |
|  | Lachnospiraceae | 17 | MR Egger | 0.04 | 0.52 | 1.04 (0.38-2.90) | 0.934 |
|  |  |  | Weighted median | 0.30 | 0.21 | 1.34 (0.89-2.04) | 0.166 |
|  |  |  | Inverse variance weighted | -0.03 | 0.21 | 0.97 (0.65-1.46) | 0.887 |
|  |  |  | Weighted mode | 0.61 | 0.47 | 1.84 (0.73-4.62) | 0.211 |
|  | Lactobacillaceae | 8 | MR Egger | 0.52 | 0.33 | 1.68 (0.88-3.24) | 0.169 |
|  |  |  | Weighted median | -0.14 | 0.17 | 0.87 (0.62-1.23) | 0.434 |
|  |  |  | Inverse variance weighted | -0.16 | 0.13 | 0.85 (0.66-1.11) | 0.230 |
|  |  |  | Weighted mode | -0.11 | 0.23 | 0.89 (0.57-1.40) | 0.642 |
|  | Methanobacteriaceae | 8 | MR Egger | 0.44 | 0.48 | 1.55 (0.61-3.94) | 0.391 |
|  |  |  | Weighted median | -0.05 | 0.12 | 0.95 (0.75-1.21) | 0.674 |
|  |  |  | Inverse variance weighted | -0.03 | 0.13 | 0.97 (0.76-1.24) | 0.810 |
|  |  |  | Weighted mode | -0.04 | 0.15 | 0.96 (0.71-1.30) | 0.805 |
|  | Oxalobacteraceae | 13 | MR Egger | -0.16 | 0.31 | 0.85 (0.47-1.55) | 0.607 |
|  |  |  | Weighted median | 0.10 | 0.11 | 1.10 (0.89-1.37) | 0.387 |
|  |  |  | Inverse variance weighted | 0.08 | 0.08 | 1.08 (0.93-1.26) | 0.330 |
|  |  |  | Weighted mode | 0.10 | 0.14 | 1.10 (0.84-1.45) | 0.495 |
|  | Pasteurellaceae | 14 | MR Egger | -0.09 | 0.24 | 0.92 (0.58-1.45) | 0.716 |
|  |  |  | Weighted median | -0.11 | 0.14 | 0.90 (0.69-1.17) | 0.427 |
|  |  |  | Inverse variance weighted | -0.16 | 0.10 | 0.86 (0.71-1.03) | 0.104 |
|  |  |  | Weighted mode | -0.11 | 0.19 | 0.90 (0.62-1.30) | 0.567 |
|  | Peptococcaceae | 9 | MR Egger | -0.02 | 0.28 | 0.98 (0.56-1.70) | 0.932 |
|  |  |  | Weighted median | 0.14 | 0.16 | 1.15 (0.85-1.56) | 0.369 |
|  |  |  | Inverse variance weighted | 0.19 | 0.12 | 1.21 (0.96-1.53) | 0.108 |
|  |  |  | Weighted mode | 0.15 | 0.19 | 1.16 (0.79-1.69) | 0.476 |
|  | Peptostreptococcaceae | 15 | MR Egger | -0.13 | 0.39 | 0.88 (0.41-1.90) | 0.751 |
|  |  |  | Weighted median | 0.18 | 0.18 | 1.20 (0.85-1.69) | 0.309 |
|  |  |  | Inverse variance weighted | 0.18 | 0.15 | 1.19 (0.89-1.59) | 0.230 |
|  |  |  | Weighted mode | -0.06 | 0.26 | 0.94 (0.56-1.58) | 0.828 |
|  | Porphyromonadaceae | 10 | MR Egger | -0.67 | 0.68 | 0.51 (0.14-1.94) | 0.355 |
|  |  |  | Weighted median | -0.20 | 0.25 | 0.82 (0.50-1.33) | 0.419 |
|  |  |  | Inverse variance weighted | -0.17 | 0.17 | 0.84 (0.60-1.19) | 0.328 |
|  |  |  | Weighted mode | -0.28 | 0.31 | 0.76 (0.41-1.40) | 0.398 |
|  | Prevotellaceae | 12 | MR Egger | -0.85 | 0.45 | 0.43 (0.18-1.03) | 0.087 |
|  |  |  | Weighted median | -0.16 | 0.22 | 0.85 (0.56-1.30) | 0.457 |
|  |  |  | Inverse variance weighted | -0.07 | 0.15 | 0.94 (0.69-1.27) | 0.665 |
|  |  |  | Weighted mode | -0.41 | 0.34 | 0.66 (0.34-1.30) | 0.259 |
|  | Rhodospirillaceae | 11 | MR Egger | -0.05 | 0.95 | 0.95 (0.15-6.12) | 0.961 |
|  |  |  | Weighted median | -0.11 | 0.18 | 0.90 (0.63-1.29) | 0.556 |
|  |  |  | Inverse variance weighted | -0.05 | 0.16 | 0.95 (0.69-1.31) | 0.766 |
|  |  |  | Weighted mode | -0.33 | 0.32 | 0.72 (0.38-1.36) | 0.334 |
|  | Rikenellaceae.id | 17 | MR Egger | -0.06 | 0.37 | 0.94 (0.45-1.95) | 0.872 |
|  |  |  | Weighted median | 0.12 | 0.18 | 1.13 (0.79-1.60) | 0.507 |
|  |  |  | Inverse variance weighted | 0.16 | 0.13 | 1.17 (0.91-1.51) | 0.220 |
|  |  |  | Weighted mode | 0.02 | 0.24 | 1.02 (0.64-1.63) | 0.941 |
|  | Ruminococcaceae | 6 | MR Egger | -0.60 | 0.62 | 0.55 (0.16-1.86) | 0.391 |
|  |  |  | Weighted median | -0.24 | 0.26 | 0.79 (0.48-1.31) | 0.355 |
|  |  |  | Inverse variance weighted | -0.26 | 0.20 | 0.77 (0.52-1.13) | 0.186 |
|  |  |  | Weighted mode | -0.25 | 0.29 | 0.78 (0.44-1.37) | 0.419 |
|  | Streptococcaceae | 10 | MR Egger | 1.43 | 0.84 | 4.19 (0.81-21.57) | 0.125 |
|  |  |  | Weighted median | 0.06 | 0.26 | 1.06 (0.64-1.78) | 0.811 |
|  |  |  | Inverse variance weighted | 0.08 | 0.24 | 1.09 (0.68-1.73) | 0.731 |
|  |  |  | Weighted mode | -0.04 | 0.36 | 0.97 (0.48-1.96) | 0.925 |
|  | Unknown family (ID: 1000001214) | 7 | MR Egger | -0.05 | 0.48 | 0.95 (0.37-2.44) | 0.924 |
|  |  |  | Weighted median | -0.08 | 0.15 | 0.92 (0.69-1.24) | 0.584 |
|  |  |  | Inverse variance weighted | -0.01 | 0.12 | 0.99 (0.79-1.25) | 0.945 |
|  |  |  | Weighted mode | -0.11 | 0.17 | 0.90 (0.64-1.25) | 0.544 |
|  | Unknown family (ID: 1000005471) | 12 | MR Egger | -0.29 | 0.43 | 0.75 (0.32-1.73) | 0.511 |
|  |  |  | Weighted median | -0.06 | 0.18 | 0.94 (0.66-1.34) | 0.729 |
|  |  |  | Inverse variance weighted | -0.05 | 0.13 | 0.95 (0.73-1.23) | 0.691 |
|  |  |  | Weighted mode | 0.05 | 0.30 | 1.05 (0.58-1.90) | 0.878 |
|  | Unknown family (ID: 1000006161) | 12 | MR Egger | 0.52 | 0.46 | 1.68 (0.69-4.10) | 0.283 |
|  |  |  | Weighted median | 0.09 | 0.13 | 1.09 (0.84-1.42) | 0.500 |
|  |  |  | Inverse variance weighted | 0.04 | 0.11 | 1.04 (0.83-1.30) | 0.743 |
|  |  |  | Weighted mode | 0.14 | 0.22 | 1.16 (0.74-1.79) | 0.531 |
|  | Veillonellaceae | 17 | MR Egger | 0.41 | 0.22 | 1.51 (0.99-2.31) | 0.075 |
|  |  |  | Weighted median | -0.02 | 0.16 | 0.98 (0.72-1.34) | 0.919 |
|  |  |  | Inverse variance weighted | 0.03 | 0.11 | 1.03 (0.84-1.27) | 0.746 |
|  |  |  | Weighted mode | 0.40 | 0.25 | 1.49 (0.91-2.45) | 0.133 |
|  | Verrucomicrobiaceae | 11 | MR Egger | 0.64 | 0.62 | 1.90 (0.57-6.38) | 0.324 |
|  |  |  | Weighted median | -0.13 | 0.22 | 0.88 (0.57-1.34) | 0.542 |
|  |  |  | Inverse variance weighted | 0.10 | 0.18 | 1.10 (0.78-1.56) | 0.579 |
|  |  |  | Weighted mode | -0.23 | 0.37 | 0.79 (0.38-1.63) | 0.544 |
|  | Victivallaceae | 12 | MR Egger | 0.18 | 0.42 | 1.20 (0.53-2.74) | 0.672 |
|  |  |  | Weighted median | -0.07 | 0.11 | 0.94 (0.75-1.17) | 0.562 |
|  |  |  | Inverse variance weighted | -0.12 | 0.09 | 0.89 (0.74-1.07) | 0.209 |
|  |  |  | Weighted mode | -0.01 | 0.14 | 0.99 (0.75-1.31) | 0.945 |
| Genus | Clostridiuminnocuum | 7 | MR Egger | -0.29 | 0.61 | 0.75 (0.23-2.47) | 0.654 |
|  |  |  | Weighted median | 0.12 | 0.15 | 1.13 (0.85-1.51) | 0.402 |
|  |  |  | Inverse variance weighted | 0.10 | 0.12 | 1.10 (0.88-1.38) | 0.399 |
|  |  |  | Weighted mode | 0.18 | 0.20 | 1.20 (0.80-1.78) | 0.407 |
|  | Eubacteriumbrachy | 10 | MR Egger | -0.02 | 0.39 | 0.98 (0.46-2.11) | 0.969 |
|  |  |  | Weighted median | 0.01 | 0.13 | 1.01 (0.78-1.29) | 0.965 |
|  |  |  | Inverse variance weighted | -0.11 | 0.10 | 0.89 (0.74-1.08) | 0.240 |
|  |  |  | Weighted mode | 0.08 | 0.20 | 1.08 (0.73-1.60) | 0.713 |
|  | Eubacteriumcoprostanoligenes | 11 | MR Egger | -0.01 | 0.92 | 0.99 (0.17-5.98) | 0.995 |
|  |  |  | Weighted median | -0.11 | 0.29 | 0.90 (0.51-1.58) | 0.709 |
|  |  |  | Inverse variance weighted | 0.06 | 0.20 | 1.06 (0.72-1.56) | 0.765 |
|  |  |  | Weighted mode | -0.37 | 0.46 | 0.69 (0.28-1.70) | 0.436 |
|  | Eubacteriumeligens | 7 | MR Egger | -0.09 | 0.89 | 0.92 (0.16-5.21) | 0.926 |
|  |  |  | Weighted median | -0.12 | 0.28 | 0.89 (0.51-1.54) | 0.672 |
|  |  |  | Inverse variance weighted | -0.33 | 0.21 | 0.72 (0.48-1.09) | 0.124 |
|  |  |  | Weighted mode | -0.01 | 0.35 | 0.99 (0.50-1.96) | 0.970 |
|  | Eubacteriumfissicatena | 9 | MR Egger | 0.13 | 0.56 | 1.14 (0.38-3.39) | 0.820 |
|  |  |  | Weighted median | 0.10 | 0.12 | 1.11 (0.87-1.41) | 0.422 |
|  |  |  | Inverse variance weighted | 0.19 | 0.10 | 1.21 (0.99-1.47) | 0.053 |
|  |  |  | Weighted mode | 0.12 | 0.17 | 1.12 (0.80-1.58) | 0.525 |
|  | Eubacteriumhallii | 13 | MR Egger | -0.31 | 0.51 | 0.73 (0.27-1.99) | 0.557 |
|  |  |  | Weighted median | 0.18 | 0.23 | 1.20 (0.77-1.86) | 0.426 |
|  |  |  | Inverse variance weighted | 0.22 | 0.24 | 1.25 (0.78-2.01) | 0.358 |
|  |  |  | Weighted mode | -0.05 | 0.30 | 0.95 (0.53-1.72) | 0.877 |
|  | Eubacteriumnodatum | 9 | MR Egger | 0.21 | 0.49 | 1.23 (0.47-3.22) | 0.680 |
|  |  |  | Weighted median | -0.01 | 0.13 | 0.99 (0.77-1.28) | 0.947 |
|  |  |  | Inverse variance weighted | -0.09 | 0.10 | 0.91 (0.75-1.12) | 0.376 |
|  |  |  | Weighted mode | 0.03 | 0.22 | 1.03 (0.68-1.57) | 0.890 |
|  | Eubacteriumoxidoreducens | 4 | MR Egger | -0.02 | 0.67 | 0.98 (0.26-3.63) | 0.979 |
|  |  |  | Weighted median | -0.01 | 0.18 | 0.99 (0.70-1.41) | 0.974 |
|  |  |  | Inverse variance weighted | -0.01 | 0.21 | 0.99 (0.66-1.49) | 0.974 |
|  |  |  | Weighted mode | 0.01 | 0.20 | 1.01 (0.68-1.51) | 0.961 |
|  | Eubacteriumrectale | 11 | MR Egger | -0.45 | 0.40 | 0.64 (0.29-1.41) | 0.299 |
|  |  |  | Weighted median | 0.01 | 0.20 | 1.01 (0.68-1.50) | 0.970 |
|  |  |  | Inverse variance weighted | -0.07 | 0.15 | 0.94 (0.69-1.26) | 0.661 |
|  |  |  | Weighted mode | 0.02 | 0.26 | 1.02 (0.61-1.71) | 0.935 |
|  | Eubacteriumruminantium | 17 | MR Egger | 0.29 | 0.44 | 1.33 (0.56-3.15) | 0.527 |
|  |  |  | Weighted median | 0.11 | 0.13 | 1.11 (0.86-1.44) | 0.430 |
|  |  |  | Inverse variance weighted | 0.08 | 0.11 | 1.09 (0.88-1.34) | 0.442 |
|  |  |  | Weighted mode | 0.14 | 0.22 | 1.15 (0.75-1.77) | 0.526 |
|  | Eubacteriumventriosum | 13 | MR Egger | 0.51 | 0.87 | 1.66 (0.30-9.08) | 0.569 |
|  |  |  | Weighted median | 0.17 | 0.21 | 1.19 (0.78-1.80) | 0.420 |
|  |  |  | Inverse variance weighted | 0.27 | 0.19 | 1.31 (0.89-1.91) | 0.166 |
|  |  |  | Weighted mode | 0.19 | 0.29 | 1.22 (0.68-2.16) | 0.520 |
|  | Eubacteriumxylanophilum | 11 | MR Egger | 0.36 | 0.63 | 1.44 (0.41-4.98) | 0.581 |
|  |  |  | Weighted median | -0.35 | 0.23 | 0.71 (0.45-1.11) | 0.131 |
|  |  |  | Inverse variance weighted | -0.06 | 0.18 | 0.95 (0.67-1.34) | 0.758 |
|  |  |  | Weighted mode | -0.52 | 0.46 | 0.59 (0.24-1.45) | 0.280 |
|  | Ruminococcusgauvreauii | 10 | MR Egger | -2.07 | 3.07 | 0.13 (0.01-52.05) | 0.520 |
|  |  |  | Weighted median | -0.19 | 0.23 | 0.83 (0.52-1.31) | 0.421 |
|  |  |  | Inverse variance weighted | 0.59 | 0.65 | 1.81 (0.51-6.45) | 0.360 |
|  |  |  | Weighted mode | -0.27 | 0.29 | 0.77 (0.44-1.34) | 0.375 |
|  | Ruminococcusgnavus | 13 | MR Egger | 0.38 | 0.50 | 1.46 (0.55-3.84) | 0.464 |
|  |  |  | Weighted median | 0.01 | 0.14 | 1.01 (0.77-1.33) | 0.948 |
|  |  |  | Inverse variance weighted | 0.05 | 0.10 | 1.05 (0.85-1.28) | 0.663 |
|  |  |  | Weighted mode | -0.07 | 0.22 | 0.94 (0.61-1.44) | 0.769 |
|  | Ruminococcustorques | 9 | MR Egger | -0.62 | 0.63 | 0.54 (0.16-1.85) | 0.358 |
|  |  |  | Weighted median | 0.10 | 0.24 | 1.10 (0.69-1.75) | 0.679 |
|  |  |  | Inverse variance weighted | 0.14 | 0.24 | 1.16 (0.73-1.84) | 0.542 |
|  |  |  | Weighted mode | -0.04 | 0.35 | 0.96 (0.49-1.91) | 0.919 |
|  | Actinomyces | 5 | MR Egger | -0.13 | 0.47 | 0.88 (0.35-2.19) | 0.798 |
|  |  |  | Weighted median | 0.06 | 0.22 | 1.06 (0.69-1.63) | 0.785 |
|  |  |  | Inverse variance weighted | 0.06 | 0.17 | 1.06 (0.76-1.47) | 0.725 |
|  |  |  | Weighted mode | 0.03 | 0.26 | 1.04 (0.62-1.74) | 0.902 |
|  | Adlercreutzia | 8 | MR Egger | -0.66 | 0.93 | 0.52 (0.08-3.21) | 0.506 |
|  |  |  | Weighted median | -0.13 | 0.19 | 0.88 (0.61-1.27) | 0.500 |
|  |  |  | Inverse variance weighted | -0.11 | 0.19 | 0.90 (0.62-1.29) | 0.562 |
|  |  |  | Weighted mode | -0.10 | 0.23 | 0.90 (0.58-1.42) | 0.672 |
|  | Akkermansia | 11 | MR Egger | 0.64 | 0.62 | 1.90 (0.57-6.36) | 0.327 |
|  |  |  | Weighted median | -0.13 | 0.21 | 0.88 (0.58-1.32) | 0.529 |
|  |  |  | Inverse variance weighted | 0.10 | 0.18 | 1.10 (0.78-1.56) | 0.579 |
|  |  |  | Weighted mode | -0.23 | 0.35 | 0.79 (0.40-1.57) | 0.523 |
|  | Alistipes | 11 | MR Egger | 0.17 | 0.90 | 1.19 (0.20-6.94) | 0.851 |
|  |  |  | Weighted median | 0.34 | 0.26 | 1.40 (0.84-2.35) | 0.196 |
|  |  |  | Inverse variance weighted | 0.25 | 0.21 | 1.29 (0.86-1.93) | 0.225 |
|  |  |  | Weighted mode | 0.37 | 0.45 | 1.45 (0.60-3.50) | 0.432 |
|  | Allisonella | 12 | MR Egger | -0.10 | 0.41 | 0.91 (0.40-2.04) | 0.818 |
|  |  |  | Weighted median | 0.07 | 0.10 | 1.07 (0.88-1.30) | 0.509 |
|  |  |  | Inverse variance weighted | 0.03 | 0.07 | 1.03 (0.90-1.19) | 0.624 |
|  |  |  | Weighted mode | 0.07 | 0.15 | 1.07 (0.79-1.45) | 0.677 |
|  | Alloprevotella | 8 | MR Egger | 0.40 | 0.53 | 1.49 (0.53-4.21) | 0.477 |
|  |  |  | Weighted median | 0.08 | 0.12 | 1.08 (0.85-1.37) | 0.518 |
|  |  |  | Inverse variance weighted | 0.05 | 0.09 | 1.06 (0.89-1.26) | 0.547 |
|  |  |  | Weighted mode | 0.16 | 0.17 | 1.17 (0.84-1.63) | 0.373 |
|  | Anaerofilum | 11 | MR Egger | 1.29 | 0.87 | 3.64 (0.66-20.13) | 0.172 |
|  |  |  | Weighted median | -0.13 | 0.16 | 0.88 (0.64-1.20) | 0.412 |
|  |  |  | Inverse variance weighted | -0.10 | 0.18 | 0.90 (0.63-1.28) | 0.561 |
|  |  |  | Weighted mode | -0.06 | 0.25 | 0.94 (0.57-1.55) | 0.82 |
|  | Anaerostipes | 14 | MR Egger | 0.42 | 0.53 | 1.51 (0.54-4.28) | 0.449 |
|  |  |  | Weighted median | -0.30 | 0.21 | 0.74 (0.49-1.11) | 0.147 |
|  |  |  | Inverse variance weighted | -0.08 | 0.15 | 0.92 (0.69-1.23) | 0.589 |
|  |  |  | Weighted mode | -0.39 | 0.37 | 0.68 (0.33-1.40) | 0.310 |
|  | Anaerotruncus | 8 | MR Egger | 0.97 | 0.79 | 2.63 (0.56-12.32) | 0.265 |
|  |  |  | Weighted median | -0.14 | 0.29 | 0.87 (0.49-1.53) | 0.627 |
|  |  |  | Inverse variance weighted | -0.10 | 0.23 | 0.90 (0.58-1.42) | 0.660 |
|  |  |  | Weighted mode | -0.08 | 0.40 | 0.92 (0.42-2.03) | 0.841 |
|  | Bacteroides | 12 | MR Egger | -0.44 | 1.07 | 0.65 (0.08-5.24) | 0.691 |
|  |  |  | Weighted median | -0.21 | 0.25 | 0.81 (0.49-1.33) | 0.407 |
|  |  |  | Inverse variance weighted | -0.02 | 0.20 | 0.98 (0.66-1.45) | 0.902 |
|  |  |  | Weighted mode | -0.46 | 0.48 | 0.63 (0.25-1.60) | 0.353 |
|  | Barnesiella | 10 | MR Egger | -0.64 | 0.81 | 0.53 (0.11-2.59) | 0.455 |
|  |  |  | Weighted median | 0.08 | 0.23 | 1.08 (0.68-1.71) | 0.749 |
|  |  |  | Inverse variance weighted | 0.11 | 0.25 | 1.11 (0.68-1.83) | 0.669 |
|  |  |  | Weighted mode | -0.53 | 0.50 | 0.59 (0.22-1.57) | 0.315 |
|  | Bifidobacterium | 14 | MR Egger | -0.23 | 0.31 | 0.79 (0.43-1.47) | 0.476 |
|  |  |  | Weighted median | 0.18 | 0.16 | 1.20 (0.87-1.65) | 0.269 |
|  |  |  | Inverse variance weighted | -0.01 | 0.12 | 0.99 (0.79-1.26) | 0.985 |
|  |  |  | Weighted mode | 0.20 | 0.28 | 1.22 (0.70- 2.12) | 0.502 |
|  | Bilophila | 12 | MR Egger | -0.04 | 0.54 | 0.96 (0.33-2.74) | 0.935 |
|  |  |  | Weighted median | -0.27 | 0.19 | 0.76 (0.52-1.11) | 0.156 |
|  |  |  | Inverse variance weighted | -0.19 | 0.14 | 0.83 (0.63-1.09) | 0.187 |
|  |  |  | Weighted mode | -0.50 | 0.31 | 0.61 (0.33-1.11) | 0.134 |
|  | Blautia | 12 | MR Egger | 0.01 | 0.38 | 1.01 (0.48-2.12) | 0.985 |
|  |  |  | Weighted median | 0.17 | 0.21 | 1.19 (0.80-1.78) | 0.396 |
|  |  |  | Inverse variance weighted | 0.02 | 0.17 | 1.02 (0.73-1.42) | 0.914 |
|  |  |  | Weighted mode | 0.25 | 0.28 | 1.28 (0.74-2.20) | 0.394 |
|  | Butyricicoccus | 9 | MR Egger | -0.45 | 0.38 | 0.63 (0.30-1.34) | 0.273 |
|  |  |  | Weighted median | -0.04 | 0.22 | 0.96 (0.63-1.47) | 0.859 |
|  |  |  | Inverse variance weighted | 0.16 | 0.23 | 1.18 (0.75-1.85) | 0.474 |
|  |  |  | Weighted mode | -0.14 | 0.23 | 0.87 (0.55-1.36) | 0.559 |
|  | Butyricimonas | 10 | MR Egger | 0.30 | 0.53 | 1.36 (0.48-3.80) | 0.579 |
|  |  |  | Weighted median | -0.28 | 0.19 | 0.75 (0.52-1.10) | 0.138 |
|  |  |  | Inverse variance weighted | -0.09 | 0.15 | 0.91 (0.68-1.22) | 0.528 |
|  |  |  | Weighted mode | -0.32 | 0.30 | 0.73 (0.41-1.29) | 0.306 |
|  | Butyrivibrio | 10 | MR Egger | 0.46 | 0.48 | 1.59 (0.62-4.08) | 0.365 |
|  |  |  | Weighted median | -0.11 | 0.12 | 0.90 (0.71-1.14) | 0.374 |
|  |  |  | Inverse variance weighted | 0.00 | 0.10 | 1.00 (0.82-1.23) | 0.970 |
|  |  |  | Weighted mode | -0.19 | 0.18 | 0.83 (0.58-1.17) | 0.316 |
|  | CandidatusSoleaferrea | 9 | MR Egger | 0.68 | 0.50 | 1.98 (0.75-5.23) | 0.213 |
|  |  |  | Weighted median | 0.23 | 0.15 | 1.26 (0.94-1.68) | 0.120 |
|  |  |  | Inverse variance weighted | 0.21 | 0.11 | 1.23 (0.99-1.54) | 0.067 |
|  |  |  | Weighted mode | 0.28 | 0.21 | 1.32 (0.87-2.00) | 0.225 |
|  | Catenibacterium | 5 | MR Egger | -0.76 | 0.75 | 0.47 (0.11-2.03) | 0.387 |
|  |  |  | Weighted median | -0.11 | 0.17 | 0.90 (0.64-1.26) | 0.529 |
|  |  |  | Inverse variance weighted | -0.12 | 0.13 | 0.89 (0.68-1.16) | 0.377 |
|  |  |  | Weighted mode | -0.07 | 0.24 | 0.93 (0.59-1.47) | 0.769 |
|  | ChristensenellaceaeR.7 | 9 | MR Egger | 0.04 | 0.84 | 1.04 (0.20-5.39) | 0.961 |
|  |  |  | Weighted median | 0.21 | 0.26 | 1.24 (0.74-2.06) | 0.412 |
|  |  |  | Inverse variance weighted | 0.05 | 0.22 | 1.05 (0.68-1.63) | 0.833 |
|  |  |  | Weighted mode | 0.19 | 0.34 | 1.20 (0.61-2.36) | 0.604 |
|  | Clostridiumsensustricto1 | 8 | MR Egger | 0.25 | 0.33 | 1.28 (0.67-2.45) | 0.485 |
|  |  |  | Weighted median | -0.09 | 0.20 | 0.92 (0.61-1.37) | 0.678 |
|  |  |  | Inverse variance weighted | -0.16 | 0.16 | 0.85 (0.62-1.17) | 0.315 |
|  |  |  | Weighted mode | -0.09 | 0.19 | 0.92 (0.63-1.34) | 0.670 |
|  | Collinsella | 7 | MR Egger | -0.54 | 1.27 | 0.58 (0.05-7.00) | 0.687 |
|  |  |  | Weighted median | -0.10 | 0.30 | 0.91 (0.51-1.63) | 0.750 |
|  |  |  | Inverse variance weighted | 0.10 | 0.30 | 1.10 (0.61-2.00) | 0.752 |
|  |  |  | Weighted mode | 0.01 | 0.37 | 1.01 (0.49-2.09) | 0.980 |
|  | Coprobacter | 12 | MR Egger | -0.01 | 0.38 | 0.99 (0.47-2.08) | 0.975 |
|  |  |  | Weighted median | -0.26 | 0.13 | 0.77 (0.59-0.99) | 0.043 |
|  |  |  | Inverse variance weighted | -0.25 | 0.10 | 0.78 (0.64-0.95) | 0.014 |
|  |  |  | Weighted mode | -0.40 | 0.24 | 0.67 (0.42-1.07) | 0.121 |
|  | Coprococcus1 | 12 | MR Egger | 0.51 | 0.48 | 1.66 (0.64-4.29) | 0.320 |
|  |  |  | Weighted median | 0.05 | 0.21 | 1.05 (0.70-1.59) | 0.804 |
|  |  |  | Inverse variance weighted | -0.02 | 0.15 | 0.98 (0.73-1.30) | 0.875 |
|  |  |  | Weighted mode | 0.17 | 0.25 | 1.18 (0.73-1.91) | 0.513 |
|  | Coprococcus2 | 7 | MR Egger | 0.20 | 0.79 | 1.22 (0.26-5.75) | 0.815 |
|  |  |  | Weighted median | -0.30 | 0.23 | 0.74 (0.48-1.16) | 0.190 |
|  |  |  | Inverse variance weighted | -0.18 | 0.18 | 0.84 (0.59-1.18) | 0.314 |
|  |  |  | Weighted mode | -0.35 | 0.32 | 0.70 (0.38-1.31) | 0.310 |
|  | Coprococcus3 | 10 | MR Egger | 0.41 | 0.61 | 1.51 (0.46-4.97) | 0.518 |
|  |  |  | Weighted median | -0.01 | 0.23 | 0.99 (0.63-1.56) | 0.975 |
|  |  |  | Inverse variance weighted | -0.11 | 0.17 | 0.90 (0.64-1.26) | 0.528 |
|  |  |  | Weighted mode | 0.14 | 0.35 | 1.15 (0.58-2.29) | 0.700 |
|  | DefluviitaleaceaeUCG011 | 9 | MR Egger | 0.58 | 0.47 | 1.78 (0.71-4.46) | 0.258 |
|  |  |  | Weighted median | 0.14 | 0.16 | 1.15 (0.84-1.57) | 0.386 |
|  |  |  | Inverse variance weighted | 0.10 | 0.13 | 1.11 (0.87-1.42) | 0.413 |
|  |  |  | Weighted mode | 0.12 | 0.23 | 1.12 (0.71-1.77) | 0.631 |
|  | Desulfovibrio | 9 | MR Egger | 0.16 | 0.60 | 1.17 (0.36-3.80) | 0.798 |
|  |  |  | Weighted median | -0.35 | 0.21 | 0.71 (0.47-1.06) | 0.094 |
|  |  |  | Inverse variance weighted | -0.11 | 0.15 | 0.90 (0.67-1.19) | 0.453 |
|  |  |  | Weighted mode | -0.39 | 0.31 | 0.68 (0.37-1.24) | 0.243 |
|  | Dialister | 8 | MR Egger | 1.58 | 1.24 | 4.87 (0.43-55.35) | 0.249 |
|  |  |  | Weighted median | -0.37 | 0.26 | 0.69 (0.42-1.14) | 0.144 |
|  |  |  | Inverse variance weighted | -0.16 | 0.27 | 0.85 (0.50-1.44) | 0.543 |
|  |  |  | Weighted mode | -0.76 | 0.50 | 0.47 (0.17-1.25) | 0.173 |
|  | Dorea | 12 | MR Egger | -0.01 | 0.35 | 0.99 (0.50-1.94) | 0.970 |
|  |  |  | Weighted median | -0.13 | 0.21 | 0.88 (0.58-1.33) | 0.541 |
|  |  |  | Inverse variance weighted | 0.02 | 0.15 | 1.02 (0.76-1.37) | 0.882 |
|  |  |  | Weighted mode | -0.20 | 0.26 | 0.82 (0.50-1.35) | 0.447 |
|  | Eggerthella | 10 | MR Egger | 0.55 | 0.70 | 1.73 (0.44-6.79) | 0.453 |
|  |  |  | Weighted median | 0.35 | 0.15 | 1.41 (1.05-1.90) | 0.022 |
|  |  |  | Inverse variance weighted | 0.25 | 0.15 | 1.29 (0.95-1.74) | 0.097 |
|  |  |  | Weighted mode | 0.36 | 0.18 | 1.43 (0.99-2.06) | 0.085 |
|  | Eisenbergiella | 11 | MR Egger | 0.82 | 0.82 | 2.27 (0.45-11.36) | 0.344 |
|  |  |  | Weighted median | 0.14 | 0.16 | 1.15 (0.85-1.57) | 0.367 |
|  |  |  | Inverse variance weighted | -0.03 | 0.12 | 0.97 (0.76-1.23) | 0.795 |
|  |  |  | Weighted mode | 0.23 | 0.27 | 1.26 (0.75- 2.12) | 0.407 |
|  | Enterorhabdus | 7 | MR Egger | 0.11 | 0.32 | 1.11 (0.60-2.08) | 0.748 |
|  |  |  | Weighted median | -0.04 | 0.14 | 0.96 (0.73-1.27) | 0.780 |
|  |  |  | Inverse variance weighted | 0.00 | 0.12 | 1.00 (0.80-1.26) | 0.985 |
|  |  |  | Weighted mode | -0.03 | 0.17 | 0.97 (0.70-1.34) | 0.858 |
|  | Erysipelatoclostridium | 13 | MR Egger | -0.31 | 0.69 | 0.73 (0.19-2.84) | 0.662 |
|  |  |  | Weighted median | -0.20 | 0.16 | 0.82 (0.60-1.13) | 0.223 |
|  |  |  | Inverse variance weighted | -0.15 | 0.14 | 0.86 (0.65-1.14) | 0.285 |
|  |  |  | Weighted mode | -0.14 | 0.22 | 0.87 (0.57-1.34) | 0.537 |
|  | ErysipelotrichaceaeUCG003 | 12 | MR Egger | 0.02 | 0.50 | 1.02 (0.38-2.71) | 0.975 |
|  |  |  | Weighted median | 0.23 | 0.23 | 1.26 (0.80-1.98) | 0.318 |
|  |  |  | Inverse variance weighted | 0.18 | 0.19 | 1.20 (0.83-1.73) | 0.329 |
|  |  |  | Weighted mode | 0.20 | 0.32 | 1.22 (0.65-2.28) | 0.543 |
|  | Escherichia.Shigella | 7 | MR Egger | 0.32 | 0.67 | 1.38 (0.37-5.17) | 0.652 |
|  |  |  | Weighted median | -0.40 | 0.25 | 0.67 (0.41-1.10) | 0.110 |
|  |  |  | Inverse variance weighted | -0.16 | 0.19 | 0.85 (0.59-1.23) | 0.390 |
|  |  |  | Weighted mode | -0.48 | 0.37 | 0.62 (0.30-1.27) | 0.238 |
|  | Faecalibacterium | 11 | MR Egger | 0.06 | 0.26 | 1.06 (0.64-1.76) | 0.813 |
|  |  |  | Weighted median | 0.28 | 0.18 | 1.32 (0.93-1.86) | 0.118 |
|  |  |  | Inverse variance weighted | 0.23 | 0.13 | 1.26 (0.98-1.63) | 0.075 |
|  |  |  | Weighted mode | 0.28 | 0.20 | 1.32 (0.89-1.96) | 0.200 |
|  | FamilyXIIIAD3011 (ID: 11293) | 14 | MR Egger | 0.09 | 1.20 | 1.10 (0.11-11.49) | 0.939 |
|  |  |  | Weighted median | 0.13 | 0.18 | 1.14 (0.80-1.64) | 0.463 |
|  |  |  | Inverse variance weighted | -0.49 | 0.43 | 0.61 (0.27-1.41) | 0.252 |
|  |  |  | Weighted mode | 0.15 | 0.23 | 1.16 (0.73-1.84) | 0.533 |
|  | FamilyXIIIUCG001 (ID: 11294) | 10 | MR Egger | 0.46 | 0.48 | 1.58 (0.62-4.03) | 0.369 |
|  |  |  | Weighted median | -0.18 | 0.18 | 0.83 (0.59-1.19) | 0.313 |
|  |  |  | Inverse variance weighted | -0.08 | 0.16 | 0.93 (0.67-1.28) | 0.646 |
|  |  |  | Weighted mode | -0.19 | 0.24 | 0.83 (0.52-1.32) | 0.446 |
|  | Flavonifractor | 6 | MR Egger | 1.05 | 0.78 | 2.87 (0.62-13.22) | 0.248 |
|  |  |  | Weighted median | 0.38 | 0.23 | 1.46 (0.93-2.31) | 0.101 |
|  |  |  | Inverse variance weighted | 0.20 | 0.18 | 1.22 (0.86-1.72) | 0.260 |
|  |  |  | Weighted mode | 0.45 | 0.35 | 1.57 (0.79-3.11) | 0.256 |
|  | Fusicatenibacter | 15 | MR Egger | -0.17 | 0.67 | 0.84 (0.23-3.13) | 0.799 |
|  |  |  | Weighted median | -0.16 | 0.19 | 0.86 (0.59-1.23) | 0.399 |
|  |  |  | Inverse variance weighted | -0.11 | 0.16 | 0.90 (0.65-1.24) | 0.522 |
|  |  |  | Weighted mode | -0.18 | 0.27 | 0.83 (0.49-1.42) | 0.512 |
|  | Gordonibacter | 12 | MR Egger | -0.42 | 0.33 | 0.65 (0.34-1.24) | 0.225 |
|  |  |  | Weighted median | 0.10 | 0.10 | 1.10 (0.91-1.33) | 0.316 |
|  |  |  | Inverse variance weighted | 0.07 | 0.07 | 1.08 (0.94-1.23) | 0.278 |
|  |  |  | Weighted mode | 0.10 | 0.11 | 1.11 (0.89-1.39) | 0.379 |
|  | Haemophilus | 9 | MR Egger | -0.19 | 0.44 | 0.82 (0.35-1.96) | 0.675 |
|  |  |  | Weighted median | -0.13 | 0.19 | 0.88 (0.60-1.29) | 0.515 |
|  |  |  | Inverse variance weighted | -0.15 | 0.17 | 0.86 (0.62-1.21) | 0.397 |
|  |  |  | Weighted mode | -0.11 | 0.29 | 0.89 (0.51-1.56) | 0.702 |
|  | Holdemanella | 9 | MR Egger | 0.74 | 0.49 | 2.09 (0.81-5.43) | 0.173 |
|  |  |  | Weighted median | -0.08 | 0.18 | 0.92 (0.65-1.31) | 0.651 |
|  |  |  | Inverse variance weighted | -0.09 | 0.15 | 0.91 (0.68-1.23) | 0.547 |
|  |  |  | Weighted mode | -0.03 | 0.22 | 0.97 (0.63-1.49) | 0.889 |
|  | Holdemania | 12 | MR Egger | 0.22 | 0.43 | 1.24 (0.53-2.89) | 0.626 |
|  |  |  | Weighted median | -0.08 | 0.17 | 0.93 (0.67-1.29) | 0.653 |
|  |  |  | Inverse variance weighted | 0.00 | 0.15 | 1.00 (0.75-1.33) | 0.984 |
|  |  |  | Weighted mode | -0.03 | 0.21 | 0.97 (0.64-1.48) | 0.901 |
|  | Howardella | 9 | MR Egger | 0.11 | 0.45 | 1.11 (0.46-2.68) | 0.818 |
|  |  |  | Weighted median | -0.03 | 0.12 | 0.97 (0.77-1.23) | 0.830 |
|  |  |  | Inverse variance weighted | 0.00 | 0.10 | 1.00 (0.82-1.23) | 0.995 |
|  |  |  | Weighted mode | 0.12 | 0.20 | 1.12 (0.76-1.66) | 0.577 |
|  | Hungatella | 6 | MR Egger | 0.54 | 0.90 | 1.71 (0.30-9.90) | 0.581 |
|  |  |  | Weighted median | 0.04 | 0.12 | 1.04 (0.82-1.33) | 0.749 |
|  |  |  | Inverse variance weighted | 0.03 | 0.09 | 1.03 (0.86-1.24) | 0.724 |
|  |  |  | Weighted mode | 0.04 | 0.14 | 1.04 (0.80-1.36) | 0.782 |
|  | Intestinibacter | 15 | MR Egger | -0.81 | 0.56 | 0.44 (0.15-1.34) | 0.172 |
|  |  |  | Weighted median | -0.05 | 0.18 | 0.95 (0.67-1.34) | 0.760 |
|  |  |  | Inverse variance weighted | -0.12 | 0.17 | 0.89 (0.64-1.24) | 0.482 |
|  |  |  | Weighted mode | -0.06 | 0.21 | 0.94 (0.62-1.41) | 0.760 |
|  | Intestinimonas | 18 | MR Egger | -0.02 | 0.35 | 0.98 (0.50-1.93) | 0.951 |
|  |  |  | Weighted median | -0.04 | 0.15 | 0.96 (0.71-1.30) | 0.792 |
|  |  |  | Inverse variance weighted | 0.04 | 0.13 | 1.04 (0.81-1.35) | 0.746 |
|  |  |  | Weighted mode | -0.03 | 0.24 | 0.97 (0.61-1.54) | 0.894 |
|  | Lachnoclostridium | 15 | MR Egger | 0.13 | 0.63 | 1.14 (0.33-3.91) | 0.842 |
|  |  |  | Weighted median | -0.40 | 0.22 | 0.67 (0.43-1.03) | 0.069 |
|  |  |  | Inverse variance weighted | -0.19 | 0.17 | 0.83 (0.59-1.15) | 0.260 |
|  |  |  | Weighted mode | -0.53 | 0.41 | 0.59 (0.27-1.30) | 0.212 |
|  | Lachnospira | 7 | MR Egger | -0.31 | 1.11 | 0.73 (0.08-6.48) | 0.790 |
|  |  |  | Weighted median | -0.49 | 0.26 | 0.61 (0.37-1.02) | 0.059 |
|  |  |  | Inverse variance weighted | -0.51 | 0.23 | 0.60 (0.38-0.94) | 0.027 |
|  |  |  | Weighted mode | -0.49 | 0.37 | 0.61 (0.30-1.26) | 0.231 |
|  | LachnospiraceaeFCS020 | 11 | MR Egger | 0.30 | 0.35 | 1.35 (0.68-2.70) | 0.418 |
|  |  |  | Weighted median | 0.18 | 0.17 | 1.20 (0.85-1.68) | 0.301 |
|  |  |  | Inverse variance weighted | 0.00 | 0.15 | 1.00 (0.75-1.33) | 0.979 |
|  |  |  | Weighted mode | 0.21 | 0.23 | 1.23 (0.78-1.94) | 0.396 |
|  | LachnospiraceaeNC2004 | 10 | MR Egger | -0.61 | 0.61 | 0.54 (0.17-1.79) | 0.346 |
|  |  |  | Weighted median | -0.24 | 0.15 | 0.79 (0.59-1.05) | 0.103 |
|  |  |  | Inverse variance weighted | -0.23 | 0.12 | 0.79 (0.63-1.00) | 0.052 |
|  |  |  | Weighted mode | -0.27 | 0.24 | 0.77 (0.48-1.22) | 0.291 |
|  | LachnospiraceaeND3007 | 4 | MR Egger | -6.39 | 3.97 | 0.01 (0.01-4.02) | 0.249 |
|  |  |  | Weighted median | 0.45 | 0.32 | 1.57 (0.83-2.95) | 0.165 |
|  |  |  | Inverse variance weighted | 0.26 | 0.27 | 1.29 (0.76-2.19) | 0.341 |
|  |  |  | Weighted mode | 0.50 | 0.38 | 1.65 (0.78-3.47) | 0.281 |
|  | LachnospiraceaeNK4A136 | 14 | MR Egger | -0.08 | 0.32 | 0.93 (0.50-1.72) | 0.814 |
|  |  |  | Weighted median | 0.06 | 0.18 | 1.06 (0.75-1.50) | 0.727 |
|  |  |  | Inverse variance weighted | -0.01 | 0.14 | 0.99 (0.76-1.29) | 0.917 |
|  |  |  | Weighted mode | 0.02 | 0.26 | 1.02 (0.61-1.70) | 0.935 |
|  | LachnospiraceaeUCG001 | 13 | MR Egger | 0.01 | 0.49 | 1.01 (0.39-2.61) | 0.988 |
|  |  |  | Weighted median | 0.16 | 0.15 | 1.17 (0.87-1.58) | 0.291 |
|  |  |  | Inverse variance weighted | 0.20 | 0.11 | 1.22 (0.97-1.52) | 0.087 |
|  |  |  | Weighted mode | 0.21 | 0.22 | 1.24 (0.80-1.90) | 0.352 |
|  | LachnospiraceaeUCG004 | 13 | MR Egger | -0.94 | 0.73 | 0.39 (0.09-1.63) | 0.224 |
|  |  |  | Weighted median | -0.23 | 0.23 | 0.79 (0.50-1.25) | 0.324 |
|  |  |  | Inverse variance weighted | -0.13 | 0.19 | 0.88 (0.60-1.28) | 0.489 |
|  |  |  | Weighted mode | -0.36 | 0.48 | 0.70 (0.27-1.79) | 0.467 |
|  | LachnospiraceaeUCG008 | 14 | MR Egger | -0.74 | 0.83 | 0.48 (0.09-2.44) | 0.393 |
|  |  |  | Weighted median | 0.10 | 0.14 | 1.11 (0.84-1.46) | 0.462 |
|  |  |  | Inverse variance weighted | 0.02 | 0.14 | 1.02 (0.77-1.35) | 0.885 |
|  |  |  | Weighted mode | 0.19 | 0.20 | 1.21 (0.82-1.79) | 0.350 |
|  | LachnospiraceaeUCG010 | 9 | MR Egger | -0.29 | 0.41 | 0.75 (0.34-1.67) | 0.500 |
|  |  |  | Weighted median | 0.12 | 0.22 | 1.12 (0.73-1.72) | 0.598 |
|  |  |  | Inverse variance weighted | 0.08 | 0.16 | 1.08 (0.79-1.48) | 0.638 |
|  |  |  | Weighted mode | -0.14 | 0.27 | 0.87 (0.51-1.47) | 0.618 |
|  | Lactobacillus | 10 | MR Egger | 0.54 | 0.32 | 1.72 (0.92-3.24) | 0.130 |
|  |  |  | Weighted median | -0.20 | 0.16 | 0.82 (0.59-1.13) | 0.231 |
|  |  |  | Inverse variance weighted | -0.22 | 0.12 | 0.80 (0.63-1.02) | 0.070 |
|  |  |  | Weighted mode | -0.14 | 0.22 | 0.87 (0.57-1.33) | 0.534 |
|  | Lactococcus | 11 | MR Egger | -0.31 | 0.42 | 0.74 (0.33-1.66) | 0.480 |
|  |  |  | Weighted median | 0.04 | 0.10 | 1.04 (0.85-1.27) | 0.717 |
|  |  |  | Inverse variance weighted | -0.04 | 0.08 | 0.96 (0.82-1.12) | 0.624 |
|  |  |  | Weighted mode | 0.07 | 0.15 | 1.08 (0.80-1.45) | 0.632 |
|  | Marvinbryantia | 10 | MR Egger | 0.63 | 0.85 | 1.88 (0.36-9.85) | 0.477 |
|  |  |  | Weighted median | -0.17 | 0.20 | 0.84 (0.57-1.25) | 0.397 |
|  |  |  | Inverse variance weighted | -0.09 | 0.20 | 0.91 (0.61-1.35) | 0.647 |
|  |  |  | Weighted mode | -0.16 | 0.22 | 0.86 (0.55-1.33) | 0.503 |
|  | Methanobrevibacter | 5 | MR Egger | 0.34 | 0.39 | 1.40 (0.66-3.00) | 0.445 |
|  |  |  | Weighted median | 0.01 | 0.14 | 1.01 (0.77-1.32) | 0.953 |
|  |  |  | Inverse variance weighted | 0.05 | 0.10 | 1.05 (0.86-1.29) | 0.648 |
|  |  |  | Weighted mode | -0.04 | 0.16 | 0.96 (0.70-1.31) | 0.806 |
|  | Odoribacter | 5 | MR Egger | -0.70 | 0.55 | 0.50 (0.17-1.46) | 0.294 |
|  |  |  | Weighted median | 0.01 | 0.27 | 1.01 (0.60-1.71) | 0.961 |
|  |  |  | Inverse variance weighted | 0.16 | 0.21 | 1.17 (0.78-1.76) | 0.447 |
|  |  |  | Weighted mode | -0.05 | 0.29 | 0.95 (0.54-1.67) | 0.861 |
|  | Olsenella | 9 | MR Egger | -0.30 | 0.35 | 0.74 (0.37-1.46) | 0.412 |
|  |  |  | Weighted median | -0.09 | 0.11 | 0.91 (0.73-1.14) | 0.419 |
|  |  |  | Inverse variance weighted | -0.07 | 0.09 | 0.93 (0.79-1.11) | 0.433 |
|  |  |  | Weighted mode | -0.11 | 0.13 | 0.89 (0.69-1.15) | 0.407 |
|  | Oscillibacter | 14 | MR Egger | 1.51 | 0.52 | 4.52 (1.64-12.48) | 0.013 |
|  |  |  | Weighted median | 0.13 | 0.16 | 1.14 (0.83-1.56) | 0.432 |
|  |  |  | Inverse variance weighted | 0.02 | 0.18 | 1.02 (0.71-1.44) | 0.930 |
|  |  |  | Weighted mode | 0.30 | 0.23 | 1.35 (0.86-2.10) | 0.214 |
|  | Oscillospira | 7 | MR Egger | 0.73 | 1.17 | 2.08 (0.21-20.79) | 0.560 |
|  |  |  | Weighted median | 0.14 | 0.24 | 1.15 (0.71-1.84) | 0.571 |
|  |  |  | Inverse variance weighted | 0.07 | 0.25 | 1.07 (0.65-1.75) | 0.790 |
|  |  |  | Weighted mode | 0.45 | 0.44 | 1.57 (0.66-3.75) | 0.350 |
|  | Oxalobacter | 14 | MR Egger | -0.12 | 0.34 | 0.88 (0.45-1.73) | 0.722 |
|  |  |  | Weighted median | 0.10 | 0.10 | 1.10 (0.90-1.34) | 0.336 |
|  |  |  | Inverse variance weighted | 0.11 | 0.09 | 1.12 (0.93-1.34) | 0.241 |
|  |  |  | Weighted mode | 0.11 | 0.12 | 1.11 (0.87-1.42) | 0.411 |
|  | Parabacteroides | 7 | MR Egger | 0.58 | 0.76 | 1.79 (0.41-7.86) | 0.477 |
|  |  |  | Weighted median | 0.43 | 0.26 | 1.54 (0.93-2.534) | 0.091 |
|  |  |  | Inverse variance weighted | 0.25 | 0.22 | 1.29 (0.83-1.99) | 0.256 |
|  |  |  | Weighted mode | 0.53 | 0.37 | 1.70 (0.82-3.54) | 0.206 |
|  | Paraprevotella | 16 | MR Egger | -0.79 | 0.44 | 0.45 (0.19-1.07) | 0.093 |
|  |  |  | Weighted median | 0.10 | 0.13 | 1.10 (0.85-1.43) | 0.453 |
|  |  |  | Inverse variance weighted | -0.08 | 0.10 | 0.93 (0.77-1.12) | 0.423 |
|  |  |  | Weighted mode | 0.13 | 0.21 | 1.14 (0.75-1.73) | 0.549 |
|  | Parasutterella | 13 | MR Egger | -0.19 | 0.43 | 0.83 (0.36-1.93) | 0.673 |
|  |  |  | Weighted median | -0.16 | 0.16 | 0.85 (0.62- 1.16) | 0.311 |
|  |  |  | Inverse variance weighted | -0.13 | 0.12 | 0.88 (0.69-1.12) | 0.303 |
|  |  |  | Weighted mode | -0.22 | 0.23 | 0.80 (0.51-1.26) | 0.353 |
|  | Peptococcus | 12 | MR Egger | -0.31 | 0.50 | 0.73 (0.28-1.95) | 0.546 |
|  |  |  | Weighted median | -0.14 | 0.13 | 0.87 (0.67-1.13) | 0.294 |
|  |  |  | Inverse variance weighted | -0.11 | 0.12 | 0.90 (0.71-1.13) | 0.356 |
|  |  |  | Weighted mode | -0.10 | 0.17 | 0.91 (0.65-1.27) | 0.580 |
|  | Phascolarctobacterium |  | MR Egger | 0.31 | 0.56 | 1.36 (0.46-4.04) | 0.591 |
|  |  |  | Weighted median | -0.11 | 0.15 | 0.90 (0.66-1.22) | 0.494 |
|  |  |  | Inverse variance weighted | -0.15 | 0.11 | 0.86 (0.69-1.08) | 0.193 |
|  |  |  | Weighted mode | -0.09 | 0.19 | 0.92 (0.63-1.33) | 0.652 |
|  | Prevotella7 | 13 | MR Egger | 0.08 | 0.41 | 1.08 (0.48-2.42) | 0.856 |
|  |  |  | Weighted median | -0.06 | 0.10 | 0.94 (0.77-1.14) | 0.522 |
|  |  |  | Inverse variance weighted | -0.05 | 0.07 | 0.95 (0.82-1.09) | 0.466 |
|  |  |  | Weighted mode | -0.09 | 0.14 | 0.91 (0.69-1.20) | 0.513 |
|  | Prevotella9 | 10 | MR Egger | -0.18 | 0.41 | 0.84 (0.37-1.88) | 0.679 |
|  |  |  | Weighted median | -0.19 | 0.16 | 0.83 (0.60-1.13) | 0.234 |
|  |  |  | Inverse variance weighted | -0.18 | 0.13 | 0.83 (0.64-1.07) | 0.158 |
|  |  |  | Weighted mode | -0.17 | 0.20 | 0.84 (0.57-1.24) | 0.403 |
|  | RikenellaceaeRC9 | 5 | MR Egger | -0.08 | 0.55 | 0.92 (0.31-2.73) | 0.895 |
|  |  |  | Weighted median | 0.03 | 0.12 | 1.03 (0.82-1.29) | 0.820 |
|  |  |  | Inverse variance weighted | 0.02 | 0.09 | 1.02 (0.85-1.22) | 0.831 |
|  |  |  | Weighted mode | -0.05 | 0.13 | 0.95 (0.73-1.24) | 0.716 |
|  | Romboutsia | 14 | MR Egger | 0.08 | 0.40 | 1.08 (0.50-2.35) | 0.847 |
|  |  |  | Weighted median | -0.09 | 0.20 | 0.91 (0.62-1.34) | 0.641 |
|  |  |  | Inverse variance weighted | -0.08 | 0.15 | 0.92 (0.68-1.25) | 0.597 |
|  |  |  | Weighted mode | -0.14 | 0.21 | 0.87 (0.57-1.32) | 0.512 |
|  | Roseburia | 17 | MR Egger | -0.71 | 0.40 | 0.49 (0.22-1.07) | 0.095 |
|  |  |  | Weighted median | -0.17 | 0.21 | 0.85 (0.56-1.27) | 0.425 |
|  |  |  | Inverse variance weighted | -0.04 | 0.14 | 0.96 (0.72-1.27) | 0.774 |
|  |  |  | Weighted mode | -0.36 | 0.31 | 0.70 (0.38-1.28) | 0.258 |
|  | Ruminiclostridium5 | 9 | MR Egger | 1.36 | 0.84 | 3.92 (0.75-20.33) | 0.148 |
|  |  |  | Weighted median | -0.24 | 0.28 | 0.78 (0.45-1.36) | 0.385 |
|  |  |  | Inverse variance weighted | -0.30 | 0.22 | 0.74 (0.48-1.15) | 0.180 |
|  |  |  | Weighted mode | 0.08 | 0.44 | 1.09 (0.46-2.59) | 0.855 |
|  | Ruminiclostridium6 | 11 | MR Egger | -1.04 | 0.43 | 0.35 (0.15-0.83) | 0.040 |
|  |  |  | Weighted median | -0.37 | 0.22 | 0.69 (0.45-1.08) | 0.102 |
|  |  |  | Inverse variance weighted | -0.20 | 0.19 | 0.82 (0.56-1.19) | 0.293 |
|  |  |  | Weighted mode | -0.47 | 0.33 | 0.63 (0.33-1.21) | 0.193 |
|  | Ruminiclostridium9 | 10 | MR Egger | 0.04 | 0.80 | 1.04 (0.22-4.98) | 0.963 |
|  |  |  | Weighted median | 0.21 | 0.24 | 1.23 (0.78-1.96) | 0.372 |
|  |  |  | Inverse variance weighted | 0.27 | 0.17 | 1.31 (0.93-1.85) | 0.117 |
|  |  |  | Weighted mode | 0.21 | 0.33 | 1.23 (0.64-2.37) | 0.548 |
|  | RuminococcaceaeNK4A214 | 13 | MR Egger | 0.12 | 0.66 | 1.13 (0.31-4.08) | 0.857 |
|  |  |  | Weighted median | -0.01 | 0.20 | 0.99 (0.67-1.47) | 0.962 |
|  |  |  | Inverse variance weighted | -0.04 | 0.15 | 0.96 (0.71-1.29) | 0.783 |
|  |  |  | Weighted mode | 0.02 | 0.30 | 1.02 (0.57-1.84) | 0.943 |
|  | RuminococcaceaeUCG002 | 18 | MR Egger | 0.26 | 0.49 | 1.29 (0.49-3.41) | 0.610 |
|  |  |  | Weighted median | -0.09 | 0.20 | 0.91 (0.62-1.36) | 0.656 |
|  |  |  | Inverse variance weighted | 0.02 | 0.18 | 1.02 (0.72-1.45) | 0.903 |
|  |  |  | Weighted mode | -0.25 | 0.33 | 0.78 (0.41-1.49) | 0.457 |
|  | RuminococcaceaeUCG003 | 13 | MR Egger | 0.20 | 0.54 | 1.22 (0.42-3.55) | 0.717 |
|  |  |  | Weighted median | -0.08 | 0.19 | 0.92 (0.64-1.33) | 0.653 |
|  |  |  | Inverse variance weighted | -0.27 | 0.16 | 0.76 (0.55-1.05) | 0.096 |
|  |  |  | Weighted mode | 0.03 | 0.24 | 1.03 (0.64-1.65) | 0.902 |
|  | RuminococcaceaeUCG004 | 9 | MR Egger | -0.74 | 0.71 | 0.48 (0.12-1.90) | 0.328 |
|  |  |  | Weighted median | -0.27 | 0.18 | 0.76 (0.53-1.09) | 0.139 |
|  |  |  | Inverse variance weighted | -0.20 | 0.14 | 0.82 (0.61-1.08) | 0.159 |
|  |  |  | Weighted mode | -0.43 | 0.30 | 0.65 (0.36-1.17) | 0.192 |
|  | RuminococcaceaeUCG005 | 12 | MR Egger | -0.59 | 0.60 | 0.55 (0.17-1.81) | 0.352 |
|  |  |  | Weighted median | -0.22 | 0.24 | 0.80 (0.50-1.27) | 0.347 |
|  |  |  | Inverse variance weighted | -0.04 | 0.22 | 0.96 (0.62-1.48) | 0.854 |
|  |  |  | Weighted mode | -0.34 | 0.31 | 0.71 (0.39-1.32) | 0.302 |
|  | RuminococcaceaeUCG009 | 12 | MR Egger | -0.67 | 0.65 | 0.51 (0.14-1.83) | 0.327 |
|  |  |  | Weighted median | -0.17 | 0.14 | 0.84 (0.63-1.12) | 0.233 |
|  |  |  | Inverse variance weighted | 0.00 | 0.14 | 1.00 (0.77-1.31) | 0.991 |
|  |  |  | Weighted mode | -0.25 | 0.20 | 0.78 (0.53-1.14) | 0.228 |
|  | RuminococcaceaeUCG010 | 8 | MR Egger | 0.00 | 0.61 | 1.00 (0.30-3.31) | 0.996 |
|  |  |  | Weighted median | 0.17 | 0.23 | 1.19 (0.75-1.87) | 0.460 |
|  |  |  | Inverse variance weighted | 0.09 | 0.17 | 1.09 (0.78-1.52) | 0.602 |
|  |  |  | Weighted mode | 0.20 | 0.28 | 1.22 (0.71-2.12) | 0.495 |
|  | RuminococcaceaeUCG011 | 8 | MR Egger | 0.85 | 0.52 | 2.35 (0.84-6.56) | 0.154 |
|  |  |  | Weighted median | 0.18 | 0.13 | 1.20 (0.93-1.54) | 0.164 |
|  |  |  | Inverse variance weighted | 0.13 | 0.10 | 1.14 (0.94-1.38) | 0.172 |
|  |  |  | Weighted mode | 0.26 | 0.19 | 1.29 (0.88-1.89) | 0.227 |
|  | RuminococcaceaeUCG013 | 14 | MR Egger | 0.32 | 0.43 | 1.38 (0.59-3.22) | 0.470 |
|  |  |  | Weighted median | 0.24 | 0.21 | 1.27 (0.83-1.93) | 0.268 |
|  |  |  | Inverse variance weighted | 0.04 | 0.15 | 1.04 (0.77-1.40) | 0.793 |
|  |  |  | Weighted mode | 0.30 | 0.30 | 1.35 (0.75-2.44) | 0.340 |
|  | RuminococcaceaeUCG014 | 8 | MR Egger | 0.34 | 0.75 | 1.41 (0.32-6.15) | 0.667 |
|  |  |  | Weighted median | -0.14 | 0.25 | 0.87 (0.53-1.42) | 0.572 |
|  |  |  | Inverse variance weighted | -0.01 | 0.23 | 0.99 (0.63-1.57) | 0.968 |
|  |  |  | Weighted mode | -0.14 | 0.42 | 0.87 (0.38-1.97) | 0.745 |
|  | Ruminococcus1 | 8 | MR Egger | 0.44 | 1.13 | 1.56 (0.17-14.22) | 0.709 |
|  |  |  | Weighted median | 0.18 | 0.29 | 1.20 (0.69-2.10) | 0.523 |
|  |  |  | Inverse variance weighted | 0.14 | 0.27 | 1.15 (0.68-1.94) | 0.596 |
|  |  |  | Weighted mode | 0.52 | 0.55 | 1.68 (0.58-4.92) | 0.372 |
|  | Ruminococcus2 | 15 | MR Egger | 0.04 | 0.42 | 1.04 (0.46-2.36) | 0.926 |
|  |  |  | Weighted median | 0.19 | 0.19 | 1.21 (0.83-1.76) | 0.328 |
|  |  |  | Inverse variance weighted | 0.12 | 0.15 | 1.13 (0.83-1.52) | 0.436 |
|  |  |  | Weighted mode | 0.38 | 0.29 | 1.47 (0.83-2.60) | 0.211 |
|  | Sellimonas | 9 | MR Egger | -0.13 | 0.52 | 0.88 (0.32-2.43) | 0.813 |
|  |  |  | Weighted median | 0.00 | 0.13 | 1.00 (0.78-1.29) | 0.996 |
|  |  |  | Inverse variance weighted | 0.05 | 0.12 | 1.06 (0.84-1.33) | 0.639 |
|  |  |  | Weighted mode | -0.19 | 0.25 | 0.83 (0.51-1.34) | 0.468 |
|  | Senegalimassilia | 3 | MR Egger | -0.28 | 0.59 | 0.76 (0.24-2.43) | 0.722 |
|  |  |  | Weighted median | -0.27 | 0.25 | 0.76 (0.47-1.24) | 0.274 |
|  |  |  | Inverse variance weighted | -0.30 | 0.20 | 0.74 (0.50-1.10) | 0.140 |
|  |  |  | Weighted mode | -0.25 | 0.28 | 0.78 (0.45-1.34) | 0.465 |
|  | Slackia | 9 | MR Egger | 0.20 | 0.55 | 1.22 (0.41-3.60) | 0.730 |
|  |  |  | Weighted median | -0.09 | 0.14 | 0.91 (0.70-1.19) | 0.505 |
|  |  |  | Inverse variance weighted | -0.02 | 0.11 | 0.98 (0.80-1.21) | 0.868 |
|  |  |  | Weighted mode | -0.12 | 0.18 | 0.88 (0.62-1.27) | 0.519 |
|  | Streptococcus | 13 | MR Egger | 0.60 | 0.68 | 1.82 (0.48-6.90) | 0.400 |
|  |  |  | Weighted median | 0.03 | 0.22 | 1.03 (0.67-1.60) | 0.879 |
|  |  |  | Inverse variance weighted | 0.03 | 0.16 | 1.04 (0.76-1.41) | 0.829 |
|  |  |  | Weighted mode | 0.02 | 0.32 | 1.02 (0.55-1.89) | 0.961 |
|  | Subdoligranulum | 11 | MR Egger | -0.15 | 0.47 | 0.86 (0.34-2.18) | 0.757 |
|  |  |  | Weighted median | -0.20 | 0.22 | 0.82 (0.53-1.26) | 0.358 |
|  |  |  | Inverse variance weighted | -0.25 | 0.17 | 0.78 (0.56-1.09) | 0.149 |
|  |  |  | Weighted mode | -0.29 | 0.31 | 0.75 (0.41-1.38) | 0.376 |
|  | Sutterella | 13 | MR Egger | 0.93 | 1.04 | 2.53 (0.33-19.28) | 0.391 |
|  |  |  | Weighted median | -0.15 | 0.20 | 0.86 (0.58-1.28) | 0.466 |
|  |  |  | Inverse variance weighted | -0.07 | 0.15 | 0.94 (0.70-1.25) | 0.655 |
|  |  |  | Weighted mode | -0.19 | 0.29 | 0.83 (0.47-1.45) | 0.524 |
|  | Terrisporobacter | 4 | MR Egger | 0.04 | 0.36 | 1.05 (0.51-2.13) | 0.912 |
|  |  |  | Weighted median | 0.01 | 0.18 | 1.01 (0.71-1.46) | 0.936 |
|  |  |  | Inverse variance weighted | 0.01 | 0.16 | 1.01 (0.74-1.39) | 0.928 |
|  |  |  | Weighted mode | 0.03 | 0.21 | 1.03 (0.69-1.55) | 0.891 |
|  | Turicibacter | 11 | MR Egger | 0.59 | 0.34 | 1.80 (0.93-3.50) | 0.117 |
|  |  |  | Weighted median | -0.07 | 0.15 | 0.93 (0.69-1.26) | 0.660 |
|  |  |  | Inverse variance weighted | 0.04 | 0.12 | 1.04 (0.83-1.31) | 0.743 |
|  |  |  | Weighted mode | -0.10 | 0.20 | 0.90 (0.61-1.33) | 0.617 |
|  | Tyzzerella3 | 12 | MR Egger | 0.34 | 0.43 | 1.41 (0.61-3.25) | 0.439 |
|  |  |  | Weighted median | 0.02 | 0.13 | 1.02 (0.80-1.31) | 0.866 |
|  |  |  | Inverse variance weighted | 0.05 | 0.09 | 1.05 (0.88-1.25) | 0.597 |
|  |  |  | Weighted mode | -0.14 | 0.20 | 0.87 (0.58-1.30) | 0.516 |
|  | Unknown genus (ID: 826) | 12 | MR Egger | -0.17 | 0.43 | 0.85 (0.36-1.96) | 0.705 |
|  |  |  | Weighted median | 0.08 | 0.19 | 1.08 (0.74-1.58) | 0.681 |
|  |  |  | Inverse variance weighted | 0.08 | 0.14 | 1.09 (0.83-1.43) | 0.549 |
|  |  |  | Weighted mode | 0.04 | 0.30 | 1.05 (0.59-1.86) | 0.884 |
|  | Unknown genus (ID: 959) | 5 | MR Egger | 1.08 | 2.33 | 2.93 (0.03-281.11) | 0.675 |
|  |  |  | Weighted median | 0.03 | 0.20 | 1.03 (0.70-1.54) | 0.868 |
|  |  |  | Inverse variance weighted | 0.03 | 0.21 | 1.03 (0.68-1.55) | 0.890 |
|  |  |  | Weighted mode | 0.37 | 0.34 | 1.44 (0.74-2.83) | 0.344 |
|  | Unknown genus (ID: 1868) | 15 | MR Egger | -0.24 | 0.53 | 0.79 (0.28-2.22) | 0.657 |
|  |  |  | Weighted median | 0.02 | 0.16 | 1.02 (0.75-1.38) | 0.917 |
|  |  |  | Inverse variance weighted | 0.05 | 0.15 | 1.05 (0.79-1.40) | 0.722 |
|  |  |  | Weighted mode | -0.13 | 0.25 | 0.88 (0.54-1.44) | 0.611 |
|  | Unknown genus (ID: 2001) | 11 | MR Egger | 0.13 | 0.50 | 1.13 (0.43-3.00) | 0.804 |
|  |  |  | Weighted median | 0.12 | 0.17 | 1.12 (0.81-1.56) | 0.486 |
|  |  |  | Inverse variance weighted | 0.04 | 0.13 | 1.04 (0.81-1.33) | 0.747 |
|  |  |  | Weighted mode | 0.17 | 0.26 | 1.19 (0.72-1.96) | 0.517 |
|  | Unknown genus (ID: 2041) | 10 | MR Egger | -0.33 | 0.38 | 0.72 (0.34-1.51) | 0.412 |
|  |  |  | Weighted median | -0.10 | 0.15 | 0.90 (0.68-1.20) | 0.481 |
|  |  |  | Inverse variance weighted | -0.11 | 0.12 | 0.89 (0.71-1.13) | 0.343 |
|  |  |  | Weighted mode | -0.05 | 0.22 | 0.95 (0.62-1.45) | 0.814 |
|  | Unknown genus (ID: 2071) | 13 | MR Egger | -1.55 | 0.54 | 0.21 (0.07-0.61) | 0.015 |
|  |  |  | Weighted median | -0.28 | 0.18 | 0.76 (0.53-1.08) | 0.122 |
|  |  |  | Inverse variance weighted | -0.22 | 0.15 | 0.80 (0.60-1.08) | 0.149 |
|  |  |  | Weighted mode | -0.42 | 0.27 | 0.66 (0.39-1.11) | 0.145 |
|  | Unknown genus (ID: 2755) | 11 | MR Egger | -0.64 | 0.75 | 0.53 (0.12-2.30) | 0.415 |
|  |  |  | Weighted median | -0.31 | 0.17 | 0.74 (0.53-1.03) | 0.071 |
|  |  |  | Inverse variance weighted | -0.22 | 0.15 | 0.81 (0.60-1.08) | 0.152 |
|  |  |  | Weighted mode | -0.34 | 0.28 | 0.71 (0.41-1.23) | 0.253 |
|  | Unknown genus (ID: 1000000073) | 15 | MR Egger | -0.05 | 0.35 | 0.95 (0.48-1.87) | 0.880 |
|  |  |  | Weighted median | 0.13 | 0.17 | 1.14 (0.82-1.59) | 0.442 |
|  |  |  | Inverse variance weighted | 0.06 | 0.12 | 1.06 (0.83-1.35) | 0.636 |
|  |  |  | Weighted mode | -0.36 | 0.31 | 0.70 (0.38-1.28) | 0.266 |
|  | Unknown genus (ID: 1000001215) | 7 | MR Egger | -0.05 | 0.48 | 0.95 (0.37- 2.44) | 0.924 |
|  |  |  | Weighted median | -0.08 | 0.15 | 0.92 (0.68-1.25) | 0.594 |
|  |  |  | Inverse variance weighted | -0.01 | 0.12 | 0.99 (0.79-1.25) | 0.945 |
|  |  |  | Weighted mode | -0.11 | 0.17 | 0.90 (0.64-1.26) | 0.552 |
|  | Unknown genus (ID: 1000005472) | 12 | MR Egger | -0.29 | 0.43 | 0.75 (0.32-1.73) | 0.511 |
|  |  |  | Weighted median | -0.06 | 0.19 | 0.94 (0.65-1.35) | 0.736 |
|  |  |  | Inverse variance weighted | -0.05 | 0.13 | 0.95 (0.73-1.23) | 0.691 |
|  |  |  | Weighted mode | 0.05 | 0.30 | 1.05 (0.59-1.88) | 0.877 |
|  | Unknown genus (ID: 1000005479) | 4 | MR Egger | 0.61 | 0.77 | 1.84 (0.41-8.37) | 0.511 |
|  |  |  | Weighted median | 0.21 | 0.28 | 1.23 (0.71-2.13) | 0.456 |
|  |  |  | Inverse variance weighted | 0.15 | 0.23 | 1.17 (0.74-1.83) | 0.502 |
|  |  |  | Weighted mode | 0.23 | 0.29 | 1.26 (0.72-2.23) | 0.478 |
|  | Unknown genus (ID: 1000006162) | 12 | MR Egger | 0.52 | 0.46 | 1.68 (0.69-4.10) | 0.283 |
|  |  |  | Weighted median | 0.09 | 0.13 | 1.09 (0.85-1.41) | 0.493 |
|  |  |  | Inverse variance weighted | 0.04 | 0.11 | 1.04 (0.83-1.30) | 0.743 |
|  |  |  | Weighted mode | 0.14 | 0.23 | 1.16 (0.74-1.82) | 0.542 |
|  | Veillonella | 9 | MR Egger | 0.28 | 0.68 | 1.33 (0.35-5.05) | 0.690 |
|  |  |  | Weighted median | -0.02 | 0.20 | 0.98 (0.67-1.44) | 0.922 |
|  |  |  | Inverse variance weighted | -0.04 | 0.14 | 0.96 (0.73-1.25) | 0.742 |
|  |  |  | Weighted mode | 0.14 | 0.31 | 1.15 (0.62-2.13) | 0.674 |
|  | Victivallis | 11 | MR Egger | -0.32 | 0.53 | 0.73 (0.26-2.06) | 0.563 |
|  |  |  | Weighted median | 0.11 | 0.11 | 1.11 (0.90-1.37) | 0.324 |
|  |  |  | Inverse variance weighted | 0.13 | 0.11 | 1.13 (0.92-1.40) | 0.242 |
|  |  |  | Weighted mode | 0.10 | 0.15 | 1.10 (0.83-1.48) | 0.515 |

Abbreviations: SLE, systemic lupus erythematosus; SNP, single nucleotide polymorphism; OR, odds ratio.
