# Supplementary material for: Causal Effects of Gut Microbiome on Systemic Lupus Erythematosus: A Two-Sample Mendelian Randomization Study
Source: Front Immunol. 2021 Sep 7;12:667097. doi: 10.3389/fimmu.2021.667097 (PMC8453215; doi:10.3389/fimmu.2021.667097)
Supplement: Supplementary file 8 [file Table_2.docx]

| **Supplementary Table 2. SNPs used as instrumental variables from individual bacterial abundance, the whole gut microbiome and SLE GWASs (*P*<5×10^-8^)** | | | | | | | | | | | | |
| --- | --- | --- | --- | --- | --- | --- | --- | --- | --- | --- | --- | --- |
| **Bacterial traits** | **SNP** | **Effect allele** | **Other allele** | **Gut microbiome** | | | **SLE** | | | **Proxy SNP** | **Target effect allele** | **Target other allele** |
|  |  |  |  | **Beta** | **SE** | ***P* value** | **Beta** | **SE** | ***P* value** |  |  |  |
| Total | rs11110281 | C | T | -0.13 | 0.02 | 7.34E-09 | -0.01 | 0.08 | 0.903 | rs11110288 | C | T |
|  | rs12781711 | C | T | -0.07 | 0.01 | 1.97E-08 | -0.03 | 0.03 | 0.325 | - | - | - |
|  | rs13163520 | A | G | -0.13 | 0.02 | 2.16E-08 | 0.01 | 0.06 | 0.858 | - | - | - |
|  | rs17159861 | C | T | 0.10 | 0.02 | 6.66E-09 | -0.02 | 0.05 | 0.658 | - | - | - |
|  | rs182549 | C | T | 0.11 | 0.01 | 2.45E-20 | -0.07 | 0.03 | 0.033 | - | - | - |
|  | rs2005426 | C | A | -0.16 | 0.03 | 4.71E-08 | 0.02 | 0.05 | 0.708 | - | - | - |
|  | rs35866622 | C | T | -0.06 | 0.01 | 1.93E-08 | -0.11 | 0.03 | 0.001 | - | - | - |
|  | rs4428215 | A | G | 0.13 | 0.02 | 4.57E-08 | 0.01 | 0.02 | 0.675 | - | - | - |
|  | rs602075 | A | G | 0.17 | 0.03 | 1.77E-08 | 0.06 | 0.03 | 0.059 | - | - | - |
|  | rs61841503 | A | G | 0.09 | 0.02 | 4.70E-08 | 0.07 | 0.04 | 0.093 | - | - | - |
|  | rs67476743 | G | T | 0.13 | 0.02 | 5.10E-09 | 0.05 | 0.03 | 0.099 | - | - | - |
|  | rs7211274 | C | T | -0.08 | 0.01 | 1.22E-08 | -0.01 | 0.04 | 0.819 | rs7221249 | A | G |
|  | rs7322849 | C | T | 0.11 | 0.02 | 3.29E-08 | 0.03 | 0.04 | 0.498 | - | - | - |
| *Actinobacteria* | rs182549 | C | T | 0.11 | 0.01 | 2.45E-20 | -0.07 | 0.03 | 0.033 | - | - | - |
| *Melainabacteria* | rs9864379 | C | T | -0.16 | 0.03 | 3.96E-08 | 0.01 | 0.03 | 0.766 | - | - | - |
| *Bifidobacteriaceae* | rs182549 | C | T | 0.12 | 0.01 | 3.86E-20 | -0.07 | 0.03 | 0.033 | - | - | - |
|  | rs7322849 | C | T | 0.11 | 0.02 | 3.29E-08 | 0.03 | 0.04 | 0.498 | - | - | - |
| *Streptococcaceae* | rs11110281 | C | T | -0.13 | 0.02 | 7.34E-09 | -0.01 | 0.08 | 0.903 | rs11110288 | C | T |
| *Allisonella* | rs602075 | A | G | 0.17 | 0.03 | 1.77E-08 | 0.06 | 0.03 | 0.059 | - | - | - |
| *Bifidobacterium* | rs182549 | C | T | 0.12 | 0.01 | 8.63E-21 | -0.07 | 0.03 | 0.033 | - | - | - |
|  | rs7322849 | C | T | 0.11 | 0.02 | 2.12E-08 | 0.03 | 0.04 | 0.498 | - | - | - |
| *Bifidobacteriales* | rs182549 | C | T | 0.12 | 0.01 | 3.86E-20 | -0.07 | 0.03 | 0.033 | - | - | - |
|  | rs7322849 | C | T | 0.11 | 0.02 | 3.29E-08 | 0.03 | 0.04 | 0.498 | - | - | - |

Abbreviations: SLE, systemic lupus erythematosus; SNP, single nucleotide polymorphism.
